# Supplementary material for: Untargeted analysis of the serum metabolome in cats with exocrine pancreatic insufficiency
Source: PLoS One. 2021 Sep 30;16(9):e0257856. doi: 10.1371/journal.pone.0257856 (PMC8483406; doi:10.1371/journal.pone.0257856)
Supplement: S1 File — Results of internal validation studies conducted by Metabolon Inc. (DOCX) [file pone.0257856.s001.docx]

**A Comparison of Missing Value Imputation Techniques for Untargeted LC-MS Metabolomics Data**

**Abstract**

For untargeted metabolomics data, one of the typical data processing steps includes the handling of missing values. The methods used for such handling depend on the sources of the missing values such as chromatographical issues, software processing errors, the concentrations are too low to be detected, or the metabolite may simply be not present in that sample, which one would expect from drugs and other xenobiotics. When missing values are the result of chromatographical issues or software processing errors, treating these as missing at random (MAR) using imputation approaches such as *k*-nearest neighbors (KNN) is reasonable. However, when the missing values are primarily the result of the values dropping below the limit of detection (LOD), approaches that assume MAR are not appropriate, as the data are not missing at random (NMAR), i.e., there is informative missingness. Furthermore, some techniques for the imputation of missing values rely on methods that require more samples than subjects, which is not true for a large number of metabolomics data sets. Data sets with fewer than 50 subjects but containing several hundred metabolites are fairly common.

A data set with 49 human subjects was analyzed to assess the sources of the missing values by manually integrating peaks that did not satisfy the thresholds of the integrator. In that data set, 46% of the missing peaks were recovered; for the remaining 54%, no peaks were present. Of the peaks recovered, 79% were below the original minimum observed values and another 18% were below the 25^th^ percentile of the original minimum. Of the total missing values, 98% either had no peak, were below the minimum or were below the 25^th^ percentile, which clearly indicates that the missingness is in general a result of falling below the LOD and should be not treated as MAR. Simulation studies were performed to compare various univariate imputation techniques. Then for the metabolites with no missing values in the data set (675 of the 1051), the lowest *q*% of the values were dropped in proportions seen in the full data, and p-values from the original data to the imputed data were compared from a two-sample t-test. For this comparison, the univariate, as well as multivariate imputation techniques such as KNN imputation, MICE, and random forest imputation were compared. Finally, for metabolites that had missing values, but all peaks were recovered, the same comparisons were made. Overall simple univariate imputations such as imputing with minimum, proportion of the minimum or half the minimum (per metabolite) outperformed the multivariate methods. Overall, imputing with a proportion of the minimum was the best performer, and imputing with the minimum also performed well for the important cases of lower percent missingness.

1. **Introduction**

Metabolomics data is typically represented by an *n* by *p* matrix **X**, where the rows are the observations and the columns are features (*n* observations, *p* features). For ion-centric data, the columns represent the peak areas for various retention time (RT) and mass combinations. Because of this, the same underlying metabolite may be represented multiple times by different ions. Often statistics are run and then elucidation is attempted for the most statistically significant features. For chemo-centric data, each column represents a unique metabolite. With chemo-centric, untargeted metabolomics data, the data consist of peak areas and identification is made by comparing to a library or database (confidence levels are described in (Schrimpe-Rutledge, Codreanu, Sherrod, & McLean, 2016)). Chemo-centric metabolomics data may also be targeted: each metabolite is run with a reference standard and calibration curves are fitted to determine the concentration. The focus of this paper is chemo-centric, untargeted data.

In metabolomics data, there are metabolites measured that do not have intensities reported for every sample. Strategies for handling the missing data depend on the reasons the values are missing. In particular it is important to distinguish missing completely at random (MCAR) or missing at random (MAR) from not missing at random (NMAR; also referred to as missing not a random (MNAR)), (Little & Rubin, 2002) i.e., non-informative missingness versus informative missingness. If there are random processing errors from the software or peaks that were missed because of alignment errors, then the missing values could be treated as MAR. However, there are lower limits of detection for each metabolite, so if the values fall below this threshold for a given metabolite, the missing values are NMAR. Furthermore, for pharmaceuticals and other xenobiotics the value may be missing because the metabolite is simply not present, and thus the missing values are NMAR.

There are few references that assess the actual sources of missing values in metabolomics data. The missing values were assessed in (Wei, et al., 2018) for two metabolomics data sets, and the authors found that gas-chromatography-mass spectrometry (GC-MS) data tended to be MCAR/MAR, while liquid-chromatography-mass spectrometry (LC-MS) data tended to be NMAR. A thorough characterization of the missing data in a large human plasma set (KORA), run on the Metabolon platform, was given in (Do, et al., 2018) where they found a large proportion of the missingness was due to batch/run day drop-outs. However, this data set is atypical in terms of the very large sample size (1757 samples, 516 metabolites), and this data set also had more observations than metabolites. Table 1 shows a breakdown by data set size for approximately all untargeted data sets run on the Metabolon platform from approximately January, 2017 – October 2019. From Table 1, one can see that only approx. 2% of data sets contained more than 1,000 observations, and data sets with 50 or fewer samples account for approximately 2/3 of the total. Furthermore, the samples in the KORA study were not processed in one long continuous run, but in multiple runs and then the data were combined. Additionally the data were collected on an older version of the Metabolon platform, which consisted of GC-MS and nominal mass LC-MS data, while the current version consists of accurate mass LC-MS data streams (Ford, et al., to appear), which have higher sensitivity, resulting in the detection of more metabolites and having fewer missing values. Finally the batch sizes have also increased since the time of the KORA study: in that study the average batch size was 34 samples, while currently 144 samples can be run in one instrument batch, so the majority of the data sets summarized in Table 1 can be run in one instrument batch, so run day missingness, which was a large source of missingness in that data set is not a common problem. In this paper, a data set with a more typical sample size is assessed in terms of characterizing the missing values. Then various strategies for imputing missing values for left-censored data are assessed: (1) simulation studies are performed comparing the power for the two-sample t-test under left-censoring with various univariate imputation methods; (2) univariate and multivariate imputation methods are compared based on a two-sample t-test for the complete data (metabolites with no missing values originally) with left-censoring applied to mimic the full data set; (3) the two-sample t-test results for metabolites that originally had some missing values but where all of the peaks were recovered were compared with various univariate and multivariate imputation methods.

1. **Characterization of Missing Values in a medium-sized data set**

This study was part of internal research and development project at Metabolon, Inc. Plasma samples were collected from 49 in-house volunteers and run on four different platforms. For this analysis, the data from platform is assessed (“V”). For each platform, data were collected from four chromatographical methods: negative ionization mode with reverse phase (NEG), negative ionization with HILIC chromatography, which was developed to better detect polar molecules (POL), and two positive ionization modes with reverse phase (POS POLAR, POS LIPID). Each of these will be referred to as arms of the platform. More details on the platform can be found in (Ford, et al., to appear) (Evans, et al., 2014), (DeHaven, Evans, Dai, & Lawton, 2010). Some metabolites may be resolved in some samples, but unresolved by the integrator in other samples. As part of the Metabolon workflow, the analyst manually re-adjusts such integration using a tool developed in-house, the Cross-set Integrator. For this data set, there were 1,051 metabolites reported; of these, 24 metabolites were re-integrated with the Cross-set Integrator. For this data set, there were 675 (64%) metabolites with no missing values, and approximately 85% of metabolites were present in at least 80% of the samples – see Table 2.

The extremely sparse metabolites are clearly xenobiotics: acetaminophen metabolites, ibuprofen metabolites, citalopram, hydroxybupropion, omeprazole, trazadone, diphenhydrame, fexofenadine, cetirizine are drugs; cotinine is a nicotine metabolite; daidzein is found in soy (Wishart, et al., 2018) and the sulfate is one if its metabolites in human plasma. So these missing values are NMAR. For the remaining metabolites, they may be present, but at concentrations too low to be detected. Next, the percent of samples present were computed by fed/fasting status (Table 3). From Table 3, one can clearly see there are many metabolites with clear differences in the percent of missing values. In particular, many dietary metabolites (metabolites of phenols, catechols, feruclic acid, vanillate, e.g.) (Wishart, et al., 2018) have strong differences and are present in more of the non-fasted subjects as expected. Note that filtering out those not present in at least 80% of one of the treatment conditions (Yang, Zhao, Lu, Lin, & Xu, 2015) or the even more stringent filtering of removing those metabolites not present in at least 80% of all of the samples (Bijlsma, et al., 2006) would have removed several informative metabolites from this analysis.

Other indications in the data that the missing values are NMAR is that many related metabolites are missing in many of the same samples or the missing samples correspond to the lower concentration samples of metabolites that are less sparse. This occurs even though they have different masses and may be measured from different data streams, so the agreement in missingness is not simply a product of the platform arm. Figure 1 gives the example for theanine and its metabolite, N-acetyltheanine, which is found in tea, mushrooms, and other potential dietary sources (Wishart, et al., 2018). Note: theanine was measured on POS POLAR, while N-acetyltheanine was measured on NEG. From Figure 1, one can see that all of the values missing for theanine were also missing for N-acetyltheanine, and that the remaining missing values for N-acetyltheanine, generally correspond to the lower values of theanine that were detected. Figure 2 has a comparison of the missingness for the 1-carboxyethyl amino acids sorted by the raw peaks areas for 1-carboxyethylphenylalanine. From this plot, one can see that in general the missing values for the other carboxyethyl amino acids correspond to the lower peak areas for 1-carboxyethylphenylalanine, again indicating the missing values are NMAR.

Next, to assess the sources of missingness in the data, for metabolites with missing values, the underlying data was re-examined for these samples. For a peak integrator, there is a trade-off between missing true peaks versus finding too many spurious peaks (e.g., randomly integrated background). Because of this, there may be some true peaks that did not pass the thresholds for the integrator, which include the minimum peak area, thresholds for the width, signal-to-noise ratio, and percent of the area estimated from missing scans, etc. An in-house software tool is available to recover the “lost” peaks, but for a normal project is too time-consuming and tedious to use. However, for this study, this tool was used to recover as many of the “lost” peaks as possible. There were 5135 missing peaks of which 2351 were recovered with the tool; for the remaining there were no peaks detected. Note: some of these recovered peaks may be of low-quality, so may have higher measurement error. The integrator parameter for the minimum peak threshold is set to 20,000. However, only approximately 35% of the recovered peaks have peak areas below 20,000. Some recovered peaks has areas above 1,000,000. For example, three peaks that were recovered for adrenate (22:4n6) had peak areas of 4,038,174, 3,362,582, 1,291,165 (the minimum of the peaks originally found by the software was 3,560,518) – this metabolite has high background levels, which is the probable cause of these peaks not surviving the integration thresholds, although the peak areas are fairly high. The distribution of the recovered peak areas is shown in Table 4.

The median and quartiles were computed for the metabolites based on the non-missing values. If the missing values were MAR then one would expect the distribution of the values to be centered about the median. Tables 5 and 6 show the distributions of the areas of the recovered peaks (Q1 = 25^th^ percentile, Q3 = 75^th^ percentile). From Table 5 one can see that 98% of the missing values either had no peak or had low values (below Q1). Of the peaks recovered, 96% were below Q1 (Table 6). To show these visually, the z-scores were computed on the log-transformed data based on the means and standard deviations of the peaks present (Figure 3). If the missing values were MAR, then one would expect to see large clusters of blue (the recovered peaks) on both sides of zero; however, this is clearly not the case. The pattern clearly indicates left-censoring.

Since there were still a large number of missing values where there were no peaks, we next assess a sample of these to see if it is reasonable to assume that these are also NMAR. As discussed earlier, a large number of missing values are from the sparse metabolites, which were drugs and other xenobiotics, so in these cases the absence of a peak probably corresponds to subjects where the metabolite was not present. For others, the absence of a peak does not necessarily indicate that it is not present, it may still be too low to be detected.

One possible way to assess this is to compare peak areas for the other MS ions for a given metabolite. However, each metabolite is typically quantitated on its most abundant ion, so this is not useful for the vast majority of the cases. One metabolite where this is possible is cortisol, for which a large in-source fragment, which corresponds to the loss of CH_2_O, is also detected. This fragment is often more intense than the parent. In this data set, cortisol was missing in one sample; however it was present for the in-source fragment. Regressing the parent on this fragment gives an R^2^ of approximately 0.99, indicating an excellent fit. The peak area for the in-source fragment corresponding to the missing parent ion is indeed the one with the lowest intensity – it only about 15% of the next highest sample. Thus, it is reasonable to infer that the missing value for cortisol is indeed NMAR.

Another way to assess the samples with no peaks is to compare values to platforms R, S, or T, where the values may be present. A small subset will be examined. For example, (R)-3-hydroxybutyrylcarnitine was originally missing in 24 samples, and 23 of these were recovered. All 23 recovered peaks were below the original minimum. Regressing the 48 peaks from platform V (so includes the recovered peaks) on those available from platform T (present for the missing sample on V, but missing in a different sample), the R^2^ was approximately 0.999, with a predicted peak area of 86,821, compared to the original minimum of 291,726, so although there was no peak here, the metabolite was probably present, but just LOD. Another such metabolite is 5alpha-androstan-3alpha,17beta-diol monosulfate (1) – it was missing in two samples originally and one was recovered. Regressing on platform R, R^2^ = 0.96, and the predicted value for this sample was 24,848 vs. 49,452, the original minimum, which also supports LOD missingness. 5alpha-pregnan-3beta,20beta-diol monosulfate (1) was missing in one sample that had no peak on platform V: regressing on the values from T, the R^2^ was 0.99 and the predicted value was 16,100 vs. the minimum of 71,514, indicating LOD not MAR. Some of the others had excellent R^2^, but the predicted values were negative, so had the problem of extrapolation, but do indicate that the missing values were probably LOD (11beta-hydroxyandrosterone glucuronide R^2^ = 0.98 using platforms R,S; 3-phosphoglycerate, R^2^ = 0.97; 4-acetylcatechol sulfate (1), R^2^ = 0.997; cortolone glucuronide (1), R^2^ = 0.935; glycine conjugate of C_10_H_14_O_2_ (1), R^2^ = 0.97; DAG(16:0/14:0)[2], R^2^ = 0.99; taurochenodeoxycholate, R^2^ = 0.95).

1. **Simulation Study**

**(3a) Univariate Imputation Techniques**

Next, a simulation study was performed to compare the power of the two-sample t-test for univariate imputation techniques. The data were assumed to follow a log-normal distribution. Simulations were run for n_1_=n_2_=5, 10, 20, 30, 50, and 100, where n_1_ and n_2_ are the sample sizes for each group. The standard deviation of the log-transformed data was 0.3 and the mean differences of the log-transformed data were set to 0, 0.15, 0.2, 0.25, and 0.3. The percent missingness tested included 5%, 10%, 20%, 30% … 70%, 80%. The missingness was generated by dropping the lowest *q*% of the data (e.g., for n_1_=n_2_=20, percent missing = 30%, the lowest 12 of the 40 values are dropped out). The following imputation methods were compared: (1) NONE – this leaves the missing values as null, so the t-test would exclude these observations; (2) MIN – impute the missing values with the observed minimum; (3) PMIN – impute the missing values with *p**observed minimum, where *p* is the proportion of non-missing values, e.g., if 25% of the values are missing, then the missing values are imputed with 0.75*observed minimum; (4) HALFMIN – impute the missing values with half the observed minimum; Methods (2) and (4) as well imputing 0 are common or used to compare to other methods (Richardson & Ciampi, 2003), (Hornung & Reed, 1990), (Wei, et al., 2018). Since the log-transformation is performed, imputation by 0 is not examined, but imputation with a low value is examined in the next section. (5) UNIFORM - here multiple imputation is performed by imputing from the uniform distribution (0 to the observed minimum). Five imputations are performed. (6) TRIANGULAR – impute from a triangular distribution (0 to observed minimum). Multiple imputation was performed analogously to (5). For (5) and (6), the sample means for each of the two groups were computed for each of the five sets. Then the five sample means were averaged for each group. The sample variances for the two groups were computed for each set. Then the average sample variances were computed for each group. These values were used in the t-test statistic and the corresponding Satterthwaite degrees of freedom. With multiple imputation, it is important to aggregate the results rather than average the data. With a large number of imputations, averaging the data for (5) would essentially be equivalent to imputing by half the minimum (4). Also for (5) and (6) the results are affected by the expected shape of the missing value portion, e.g., for a log-normal distribution with fewer than 50% of the values missing, a triangle can approximate the missing portion. (Hornung & Reed, 1990). The imputations were performed before the log-transformation. A total of 10,000 simulation runs were performed for each combination.

Figure 4 shows the Type I error. Most combinations are close to 0.05, the nominal level (note: there may also be some small deviations since Welch’s two-sample t-test using Satterthwaite’s degrees of freedom has an *approximate* t-distribution). For some of the combinations with small sample sizes and a high proportion of missing values, some methods have actual Type I errors below the nominal levels, resulting in the statistics that are more conservative than the nominal level.

Figure 5 shows a typical pattern in terms of power. The actual powers for these combinations are approximately 0.14, 0.29, 0.54, 0.73 for n_1_=n_2_=5, 10, 20, and 30, respectively (n_1_=n_2_=50 and 100 were not shown as the power was close to 1). When the percentage of missing values is 30% or less, PMIN and MIN are the best performing and comparable. For higher levels of missingness, HALFMIN and PMIN perform the best. Ignoring the missing values by leaving them null performs much worse than the imputed methods. The two multiple imputation methods also perform poorly compared to MIN, PMIM, and HALFMIN. In terms of the importance of the various combinations, it is more desirable to have optimal performance for those with fewer missing values as this is more common (Table 2) and one would be more confident in results that rely less on imputation (e.g., 10% missing is a more important case than 70% missing). Thus, MIN performs better than HALFMIN for the more important cases; however, PMIN performs well in both cases. The full tables for the estimated power for all the cases simulated are shown in the Appendix. All simulations and data analysis were performed with *R*, version 3.5.3 (R Core Team, 2019).

**(3b) Simulations Using Based on the Data Set**

The simulations were performed under certain conditions that may differ from true data and only addressed the univariate case. Often, to test the methods, the data without any missing values, will have various proportions randomly set to missing (Shah, et al., 2017) (Nguyen, Wang, & Carroll, 2004), (Troyanskaya, et al., 2001), (Miecznikowski, Damodaran, Sellers, & Rabin, 2010), (Wei, et al., 2018). For MCAR/MAR, one could simply choose the percent from the whole data to randomly set to missing. For NMAR, it is better to simulate the percent missingness per variable. For example, if 10% of the metabolites were missing in 20% of the samples for the full data set, then set 10% of the variables in the complete data are set to 20% missingness with a left-censoring mechanism (i.e., the lowest 20% is set to missing for each of these variables). Missingness for the 675 complete metabolites was set to the proportions missing in the entire data set for 0% to 80% missing. Let p_0_ be the original p-value for the metabolite (full data) and let p_1_ be the p-value for the imputed metabolite. Let A_0_ = -1*log(p_0_,base=10) and A_1_ = -1*log(p_1_,base=10). For each metabolite, DA = |A_0_ – A_1_| was computed. The DA values were then averaged for each percent missing in each simulation run, and then across the simulations (250 simulation runs here).

This method of simulating missingness in the complete data has the limitation that the percent missingness for one metabolite is independent of the others, which as we have seen earlier is not realistic as often related metabolites are missing in many of the same samples (theanine, N-acetyltheanine) or the missing samples correspond to lower values of a related variable (1-carboxyethylphenylalanine vs. 1-carboxyethyltyrosine). Additionally, metabolites that often have missing values may behave differently from those that often do not have missing values.

To address these limitations, the p-values are also compared for those metabolites that had missing values, but where all their missing values were recovered. There were 162 variables with missing values where all the “lost” peaks were recovered. Here the results of the two-sample t-test were compared to the imputed data vs. the data with the recovered peaks. For this analysis because the extremely sparse metabolites may cause issues for some of the multivariate algorithms that use all of the variables (whether they have missing values or not), metabolites missing in more than 80% of the samples were excluded (which were xenobiotics here, mostly drugs).

**(3b-i) Imputation Methods Assessed**

As with the simulations, (1) NONE – no imputation was performed, so the analysis is based only on the complete cases. From the univariate simulation (2) PMIN, (3) MIN, and (4) HALFMIN performed fairly well, so these methods are tested here. (5) THRESH imputes with half the minimum across the *entire* data set (Xia, Psychogios, Young, & Wishart, 2009), (Xia & Wishart, Web-based inference of biological patterns, functions and pathways from metabolomic data using MetaboAnalyst, 2011). This method and similar methods such a imputing a “small value” or “zero” across the whole data set were also examined in other works (Webb-Robertson, et al., 2015), (Di Guida, et al., 2016), (Hrydziuszko & Viant, 2012), (Shah, et al., 2017). This assumes that each metabolite has the same LOD, which is extremely dubious based on the characterization of the missing values performed earlier.

(6) RF – random forest imputation; Random Forest is an ensemble method that aggregates the predictions from classification or regression trees (Breiman, 2001). One imputation method, “missForest” imputes based on random forest *regression* (Stekhoven & Buhlmann, MissForest - non-parametric missing value imputation for mixed-type data, 2012). An example of a regression tree is the following: let *y*_1_ be the average value of *y* when *x* > 1 and let *y*_2_ be the average value of *y* when *x* < 1; the predicted value of *y* when *x* > 1 is *y*_1_ and *y*_2_ otherwise. Random forest regression aggregates the results across many such regression trees. For the imputation performed by the missForest algorithm, the missing values are initially imputed with the mean. Then the variables are imputed sequentially based on the proportion of missing values, i.e., the variable with the fewest missing values is imputed first (based on the observations without missing values for that variable), the data set is then updated with these values, then the variable with the next fewest missing is imputed based on this updated data, etc. The process is iterated until convergence. These imputations were performed with the “missForest” *R-* package (Stekhoven, 2013).

One nice property of this imputation technique is that it can be performed on the metabolites with their original raw areas – no re-scaling is needed. However, since the t-tests will be performed on the log-transformed data, the results may differ if the imputation is performed and then the log-transform applied as opposed to imputation performed on the log-transformed data. For the regression tree listed above, it has no effect on the *x*-variable since this is a monotonic transformation, hence *x* > 1 is equivalent to the rule log(*x*) > 0. However, the predicted values are affected since the average of log(*y*) is not equal to log(avg(*y*)). Because of this, the imputation was performed on the log-transformed data.

One final clarification: this imputation is different than the imputation function in the randomForest R –package (Liaw & Wiener, 2002), *rfImpute*, which was the preferred imputation method for (Gromski, et al., 2014). This imputation applies to the supervised case only, so for this data set it would use the fasted/fed status as the response variable. The missing values are first imputed with their column medians. Then random forest classification is performed. For each variable, the final imputed values are a weighted average of the observed values where the weights are the proximities. The proximity measure for two observations is the number of times both observations occur in the same terminal node of one of the classification trees divided by the total number of trees. This method of imputation is not assessed here as it is supervised and missForest is more commonly used.

(7) KNN: *k*-nearest neighbors imputation. The “neighbors” can refer to either the observations (Tutz & Ramzan, 2015), (Kowarik & Templ, 2016), (Wasito & Mirkin, 2006), or the variables (Shah, et al., 2017), (Nguyen, Wang, & Carroll, 2004) , (Troyanskaya, et al., 2001), (Di Guida, et al., 2016), (Hrydziuszko & Viant, 2012) , (Oba, et al., 2003), (Wei, et al., 2018), (Lazar, Gatto, Ferro, Bruley, & Burger, 2016). Combinations of both were assessed in (Do, et al., 2018) where KNN imputation based on the nearest observations as determined from a subset of the variables was one of the preferred methods. The imputed value can simply be the median of the values from the neighbors or a weighted average based on the inverse distances, which are defined with a metric such as the Euclidean distance.

For KNN imputation based on the *k* nearest observations, if one wants to impute the value for observation *i* and variable *j*, first compute the distance from observation *i* to all the other observations with a distance metric. The distance between two samples can be computed based on all non-missing pairs across all variables or just a subset of variables. Then the *k* observations with the smallest distance to observation *i* are determined to be the nearest neighbors. Then the imputed value is the aggregated value of variable *j* for those *k* observations such as the median or a weighted average based on the inverse distances (if D = 1/d_1_ + 1/d_2_ + … 1/d_k_, then the weights are 1/(d_1_*D), 1/(d_2_*D), …, 1/(d_k_*D)).

KNN imputation can also be based on the *k* nearest variables. For imputing observation *i*, variable *j*, one first computes the *k* variables with the smallest distance (correlation, weighted Euclidean distance, e.g.) to variable *j*. Then the imputed value is the median or weighted average of the *k* variables. Alternatively, one could find the *k* top individual regressors to variable *j* (most correlated by absolute value), then perform ordinary least-squares (OLS) separately for each of the *k* variables and then aggregate the predictions (Nguyen, Wang, & Carroll, 2004). For the weighted average, d_1_, d_2_, …, d_k_ are the estimates of σ^2^ from the regressions. The issues of re-scaling/truncation is less important for OLS since the predicted values from the regressions are used, rather than the values of the variables themselves. For the correlation distance, we considered only positive correlations since the median of these variables or linear combination is computed: d = 1 – r (if we also used negative correlations it would be 1 - |r|). For this analysis, only variables with no missing values were candidates for the neighbors when performing KNN by variable imputation.

For KNN imputation, especially imputation by variables, it is important the each variable is on the same scale. This can be achieved by computing the z-score for each variable, or simply re-scaling each variable by the median. We prefer re-scaling every column to the median to preserve the variability, and then taking the log-transformation for statistical analysis (Steuer, Morgenthal, Weckwerth, & Selbig, 2007). Either of these re-scaling techniques is completely appropriate when the data is MAR. However, as noted by (Shah, et al., 2017), this can be problematic if the missing values are left-censored since the true median (or mean) is lower than that of the observed data. To remedy this, they compute the maximum likelihood estimates for the mean and standard deviation based on the left-truncated normal distribution. Then the columns are converted to z-scores using these estimates. In the case of log-normal data, these computations should be performed on the log-transformed data. One potential problem by re-scaling in this manner occurs if the variable has a bimodal distribution, which can occur if there are two outcome groups with strong mean differences, so estimating the mean and standard deviation using all the values is problematic. As an alternative, the median is estimated assuming left-truncation, which is straight-forward when the variable is present in at least 50% of the samples (for the case where 50% of values are missing, if left-truncation is assumed, then an estimate of the median is the observed minimum). If a variable was absent in more than 50% of the samples, then maximum likelihood was used to estimate the median on the log-scale then back-transformed. Then all variables were median-scaled and log-transformations performed. The maximum likelihood estimation was performed with the *survival* *R*-package. (Therneau, 2015). For the KNN imputation itself, we wrote our own *R*-functions, rather than using one of the packages.

Many combinations of KNN imputation were tested on the median-scaled, log-transformed variables: imputation from the nearest *k* observations or nearest *k* variables; for KNN by observation, various subsets of variables were used; for KNN by variables, correlation, weighted Euclidean distance (the square root of the average of the squared differences for every pair where both measurements were not missing), or OLS was performed. For KNN by variables, analysis was performed on the median-scaled, log-transformed data with and without assuming left-truncation. The following notation is used: KNN(TYPE, k, VAR, METRIC, WEIGHTS, TRUNC), where *k* is the number of nearest neighbors, TYPE indicates whether the imputation was based on the *k* nearest observations or *k* nearest variables (“obs” or “var,” respectively), VAR are the variables used to determine the nearest neighbors for TYPE=”obs.” Values can be “all” (all variables used), “comp” (only variables without missing values), “all(v)”, v most correlated variables (by absolute value), “comp(v)” the *v* most correlated variables from those without missing values. For KNN imputation by variables this value is always “comp(k)” except only positive correlations are considered, METRIC = “EUCL” for weighted Euclidean distance, “CORR” for the most correlated variables, “OLS” for the *k* strongest regressors. WEIGHTS has values “median” or “weighted distance.” TRUNC has value “T” if the data are re-scaled data assuming left-censoring and null otherwise.

(8) MICE – Multiple Imputation by Chained Equations

The general technique is to first impute all the missing values with a value such as the column mean for the initialization step (Azur, Stuart, Frangakis, & Leaf, 2011), then to use Gibbs sampling to create successive draws for the parameters and the imputed values through chained equations (van Buuren & Groothuis-Oudshoorn, 2011). More specifically, let X_1_, X_2_, X_3_, …, X_p_ represent the columns of **X**. For each X_j_, let U_j_ consist of the observed values and let Y_j_ consist of the missing values. Let ψ_j_ represent the parameters for the predictions of variable *j* (regression coefficients and σ^2^ , e.g.). Let the subscripts *jk* indicate the jth variable at the kth iteration. The kth iteration consists of draws as the following: ψ_1k_ ~ P(ψ_1_|U_1_,X_2k_, .., X_pk_), then Y_1k_ ~ P(X_1_|U_1_,X_2k_,…, X_pk_, ψ_1k_), …, : ψ_1p_ ~ P(ψ_p_|X_1k_,X_2k_, .., U_pk_), then Y_pk_ ~ P(X_p_|X_1k_,X_2k_,…, U_p_, ψ_pk_).

The common implementation of this method is through the mice R-package. (van Buuren & Groothuis-Oudshoorn, 2011). For the initialization step, the default is to impute each column with missing data with a bootstrap sample of the observed values. Then the values are imputed successively through different methods, the most common being “norm” and “pmm.” “Norm” refers to Bayesian regression using a normal probability model. The specific algorithm is given by Algorithm 3.1 in (van Buuren S. , 2018), which also applies a ridge penalty (from the source code it appears that the default is to perform linear regression via the QR decomposition and then apply a ridge penalty if the cross-product matrix is not invertible, which will occur when there are more predictors than observations). The imputed values are the predicted values from the regression plus random noise from a normal distribution where the variance is determined from the regression. The other common method is “pmm,” which is predictive mean matching. Bayesian regression is performed as described, but the predicted values for the samples to be imputed are compared to the predicted values for the observed data for the given variable. The observations “closest” to the imputed sample are computed and then the imputed value is a random draw of these (van Buuren S. , 2018).

Running MICE on the full original data set without the extremely sparse metabolites (missing in more than 80% of the observations) with the default parameter values either crashed or produced errors. However, we were able to get the program to run successfully by changing the “predictorMatrix” argument. This is a *p* by *p* matrix where each row is a variable to be imputed and the value in column *j* is equal to 0 if variable *j* is not a predictor and equal to 1 if it is used as a predictor in the regression. The default is a matrix all 1s except along the diagonal which has 0s (variables with no missing values are skipped over when running the program). Since only a small subset of variables should be needed for each regression model, we changed this matrix from the default to include only a subset of the most correlated variables (by absolute value) as predictors for each variable with missing values (so each variable may have different predictor variables), but because of this variable selection, the variability may be underestimated. For these MICE models, *m*=5 imputations were performed. The results were aggregated as follows for the two-sample t-test: the sample means for fasted and not fasted were computed for each of the five sets. Then the five sample means were averaged for each group. The sample variances for fasted and not fasted were computed for each set. Then the average sample variances were computed for each condition. These values were used in the t-test statistic and the corresponding Satterthwaite degrees of freedom were computed.

(9) PLS – Partial Least-Squares

Let **X_c_** consist of the columns of **X** with no missing values. Here each variable with missing values is predicted based on the first component from a PLS regression with the variable to be imputed as “y” and the regressors are **X_c_**. Each predicted value (used for the imputation) is a linear combination of all the variables in **X_c_**. All of the variables were median-scaled, then log-transformed. The PLS regression is fitted on all the y values without missing values and the values in **X_c_** for those same observations. Then the missing values are predicted from the model. Note: this could also be performed in a manner similar to RF and MICE where all missing values are imputed with an initial value and then updated. For example, (Nguyen, Wang, & Carroll, 2004) perform PLS on each variable on all of the remaining variables in **X** by providing an initial imputation by KNN. However, since 64% of the variables had no missing values and we wanted to avoid imputations based on imputations from other variables, we used the only the complete variables as the regressors.

**(3b-ii) Results: Complete Case Data Simulation**

Since there were 28 combinations of KNN imputation applied to the data, we first compare these. KNN(var,10,…) was at least as good as KNN(var,20,…), so the *k*=20 cases are not displayed on the plots. Also, both median and weighted distances were used for each combination, but the results were very similar so the results for the median are not shown on the plot. From Figure 6, the worst performing methods were KNN(obs,5,all,eucl,wtd) and KNN(obs,5,comp,eucl,wtd), which is not surprising since the distance between samples was based on a large number of variables. Additionally, KNN(obs,…) cannot produce imputed values below the observed minimum, which is a definite problem for left-censored data. KNN(obs,5,all(10)) and KNN(obs,5,comp(10)) improved over the KNN(obs,…) with no variable restrictions. These performed similarly to KNN(var,10,…) . For higher percent missingness, the weighted OLS predictions performed better than the other methods that did not assume truncation. However, taking into account the left-censoring, especially with increasing missingness, was a clear improvement and were the best performers among the KNN methods. Interestingly, for low percent missing, using the correlation to define the nearest neighbors (variables) was the best performer, but with higher percent missing, using the Euclidean distance between variables performed better.

Figure 7 shows a comparison of the methods with a small subset of the KNN combinations (the best KNN(obs,…) and the two best KNN(var,…) methods). MICE was run with subsets of 10 and 20, but the results were fairly similar, so only the results for 10 are shown. From Figure 7, one can see that imputing the entire data set with an arbitrary low number (THRESH) has poor performance with low percent missingness (including just one missing value of the 49!). Not surprisingly, MICE(norm) outperformed MICE(pmm), since the latter cannot produce an imputed value lower than the observed minimum for a given metabolite. This is the same issue for RF and KNN(obs,…), which cannot produce imputed values below the minimum, so are outperformed by the other methods. Although PLS could produce imputed values below the minimum, it was one of the weaker performers. For fewer than 30% missing values, the univariate methods, MIN, PMIN, and HMIN outperform the other methods. PMIN is the strongest performer overall.

**(3b-iii) Results: Comparisons based on Metabolites with all lost peaks recovered**

As we have seen earlier, the majority of missingness can be attributed to falling below the limit of detection, but there were some small exceptions. Additionally, the metabolites that commonly have missing values may behave differently than those that routinely do not have missing values. Thus, we compare the imputation for those metabolites with missing values where all the peaks were able to be recovered: 162 metabolites. One limitation of this analysis is that the percent missing was fairly low for these metabolites before the lost peaks were recovered: 117 were present in at least 90% of the samples, 30 metabolites were present in 80-89% of the samples, while 15 metabolites were originally present in fewer than 80% of the samples.

The medians and quartiles of the absolute differences of the log10 p-values were computed. A summary is shown in Table 7. Of the multivariate methods, MICE(norm,10) was the best performer. Unlike the previous analysis, the truncation adjustment for KNN(var,…) had little improvement over the standard – this is probably because most these metabolites had low percent missing originally. Overall, the univariate methods MIN, PMIN, and HALFMIN clearly outperform the other methods and performed similarly.

1. **Discussion**

When developing strategies for handling missing values, it is important to understand the sources of the missingness. Many strategies are designed for non-informative missingness/MAR. However, these strategies can weaken the power to find the differences in the data if the missing values are NMAR. In particular, KNN(obs,…), RF, and MICE(pmm) can produce imputed values below the observed minimum for a given metabolite, which is problematic for left-censored data. For a mid-size human metabolomics data set, 98% of the missing values either had no peak or had values below the first quartile of the peaks originally found by the software, indicating left-censoring - NMAR. The LOD should be estimated separately for each metabolite, rather than estimated across the entire data set.

For the case of LOD-missingness (left-censoring), simple single imputation univariate methods such as imputing with the minimum, half minimum, or a proportion of the minimum (impute with *p***m_0_*, where *m_0_* is the observed minimum and *p* is the proportion of non-missing values) outperformed common multivariate techniques. Overall, imputing with a proportion of the minimum was the best performer, and imputing with the minimum was comparable for the cases lower percent missing, which is the more common and more important case.

1. **Bibliography**

Armitage, E. G., Godzien, J., Alonso-Herranz, V., Lopez-Gonzalvez, A., & Barbas, C. (2015). Missing value imputation strategies for metabolomics data. *Electrophoresis, 36*, 3050-3060.

Azure, M. J., Stuart, E. A., Frangakis, C., & Leaf, P. J. (2011, March). Multiple Imputation by Chained Equations: What is it and how does it work? *Int J Methods Psychiatr Res, 20*(1), 40-49. doi:doi:10.1002/mpr.329

Bijlsma, S., Bobeldijk, I., Verheij, E. R., Ramaker, R., Kochchar, S., Macdonald, I. A., . . . Smilde, A. K. (2006). Large-Scale Human Metabolomics Studies: A Strategy for Data (Pre-) Processing and Validation. *Analytical Chemistry, 78*, 567-574. doi:10.1021/ac051495j

Breiman, L. (2001). Random Forests. *Machine Learning, 45*, 5-32. doi:https://doi.org/10.1023/A:101093340

DeHaven, C., Evans, A., Dai, H., & Lawton, K. (2010). Organization of GC/MS and LC/MS metabolomics data into chemical libraries. *Journal of Cheminformatics, 2*(9). doi:10.1186/1758-2946-2-9

Di Guida, R., Engel, J., Allwood, W. J., Weber, R. J., Jones, M. R., Sommer, U., . . . Dunn, W. B. (2016). Non-targeted UHPLC-MS metabolomic data processing methods: a comparative investigation of nomralisation, missing value imputation, transformation and scaling. *Metabolomics, 12*(93). doi:10.1007/s11306-016-1030-9

Do, K. T., Wahl, S., Raffler, J., Molnos, S., Laimighofer, M., Adamski, J., . . . Krumsiek, J. (2018, October). Characterization of missing values in untargeted MS-based metabolomics data and evaluation of missing data handling strategies. *Metabolomics, 14*(10). doi:https://doi.org/10.1007/s11306-018-1420-2

Evans, A. M., Bridgewater, B. R., Mitchell, M. W., Robinson, R. J., Dai, H., Stewart, S. J., . . . Miller, L. A. (2014). High Resolution Mass Spectrometry Improves Data Quantity and Quality as Compared to Unit Mass Resolution Mass Spectrometry in High-Throughput Profiling Metabolomics. *Metabolomics, 4*(132). doi:10.4172/2153-0769.1000132

Ford, L., Kennedy, A. D., Goodman, K. D., Pappan, K. L., Evans, A. M., Miller, L. A., . . . Toal, D. R. (to appear). Precision of a Clinical Metabolomics Profling Platform for Use in the Identification of Inborn Errors of Metabolism. *Journal of Applied Medicine*.

Gromski, P. S., Xu, Y., Kotze, H. L., Correa, E., Ellis, D. L., Armitage, E. G., . . . Goodacre, R. (2014). Influence of Missing Values Substitutes on Multivariate Analysis of Metabolomic Data. *Metabolites, 4*, 433-452. doi:10.3390/metabo4020433

Hornung, R. W., & Reed, L. D. (1990). Estimation of Average Concentration in the Presence of Nondetectable Value. *Appled Occupational and Environmental Hygeine, 5*(1), 46-51. doi:https://doi.org/10.1080/1047322X.1990.10389587

Hrydziuszko, O., & Viant, M. R. (2012). Missing values in mass spectrometry based metabolomics: an undervalued step in the data processing pipeline. *Metabolomics, 8*, S161-S174. doi:10.1007/s11306-011-0366-4

Lazar, C., Gatto, L., Ferro, M., Bruley, C., & Burger, T. (2016). Accounting for the multiple natures of missing values in label-free quantitative proteomics data sets to compare imputation strategies. *Journal of Proteome Research, 15*, 1116-1125. doi:10.1021/acs.jproteome.5b00981

Liaw, A., & Wiener, M. (2002). Classification and Regression by randomForest. *R News, 2*(3), 18-22. Retrieved from https://CRAN.R-project.org/doc/Rnews/

Little, R. J., & Rubin, D. B. (2002). *Statistical Analysis with Missing Data.* Hoboken, New Jersey: John Wiley & Sons, Inc.

Miecznikowski, J. C., Damodaran, S., Sellers, K. F., & Rabin, R. A. (2010). A comparison of imputation procedures and statistical tests for the analysis of two-dimensional electrophoresis data. *Proteome Science, 8*(66). doi:10.1186/1477-5956-8-66

Nguyen, D. V., Wang, N., & Carroll, R. J. (2004). Evaluation of Mssing Value Estimation for Microarray Data. *Journal of Data Science, 2*, 347-370.

Oba, S., Sato, M.-a., Takermasa, I., Monden, M., Matsubara, K.-i., & Ishii, S. (2003). A Baysian missing value estimation method for gene expression profile data. *Bioinformatics, 19*(16), 2088-2096. doi:10.1093/bioinformatics/btg287

R Core Team. (2019). R: A language and environment for statistical computing. *R Foundation for Statistical Computing, Vienna, Austria*. Retrieved from https://www.R-project.org/

Richardson, D. B., & Ciampi, A. (2003). Effects of Exposure Measurement Error When an Exposure Variable is Constrained by a lower limit. *Journal of Epidemiology, 157*(4), 355-363. doi:10.1093/aje/kwf217

Schrimpe-Rutledge, A. C., Codreanu, S. G., Sherrod, S. D., & McLean, J. A. (2016). Untargated Metabolomics Strategies - Challenges and Emerging Directions. *Journal of the American Society for Mass Spectrometry, 27*, 1897-1905. doi:10.1007/s13361-016-1469-y

Shah, J. H., Rai, S. N., DePhilippis, A. P., Hill, B. G., Bhatnagar, A., & Brock, G. N. (2017). Distribution based nearest neighbor imputation for truncated high dimensional data with applications to pre-clinical and clinical metabolomics studies. *BMC Bioinformatics, 8*(114). doi:10.1186/s12859-017-1547-6

Stekhoven, D. J. (2013). *missForest: Nonparametric Missing Value Imputation using Random Forest, R package*.

Stekhoven, D. J., & Buhlmann, P. (2012). MissForest - non-parametric missing value imputation for mixed-type data. *Bioinformatics, 8*(1), 112-118. doi:doi:10.1093/bioinformatics/btr597

Steuer, R., Morgenthal, K., Weckwerth, W., & Selbig, J. (2007). A Gentle Guide to the Analysis of Metabolomic Data. *Methods in Molecular Biology, 358*, 105-126. doi:10.1007/978-1-59745-244-1_7

Therneau, T. M. (2015). A Package for Survival Analysis. Retrieved from https://CRAN.R-project.org/package=survival

Troyanskaya, O., Cantor, M., Sherlock, G., Brown, P., Hastie, T., Tibshirani, R., . . . Altman, R. B. (2001). Missing value estimation methods for DNA microarrays. *Bioinformatics, 17*(6), 520-525. doi:https://doi.org/10.1093/bioinformatics/17.6.520

Tutz, G., & Ramzan, S. (2015). Improved methods for the imputation of missing data by nearest neighbor methods. *Computation Statistics and Data Analysis, 90*, 84-99. doi:10.1016/j.csda.2015.04.009

van Buuren, S. (2018). *Flexible Imputation of Missing Data* (Second ed.). Boca Raton, Florida: CRC/Chapman & Hall.

van Buuren, S., & Groothuis-Oudshoorn, K. (2011). Mice: multivariate imputation by chained equations in R. *Journal of Statistical Software, 45*(3), 1-67. Retrieved from https://www.jstatsoft.org/article/view/v045i03

Wasito, I., & Mirkin, B. (2006). Nearest neighbors in least-squares data imputation algorithms with different missing patterns. *Computational Statistics & Data Analysis, 50*, 926-949. doi:10.1016/j.csda.2004.11.009

Webb-Robertson, B.-J. M., Wiberg, H. K., Matzke, M. M., Brown, J. N., Wang, J., McDermott, J. E., . . . Waters, K. M. (2015). Review, Evaluation and Discussion of the Challanges of Missing Value Imputation for Mass Spectrometry Label-Free Global Proteomics. *Journal of Proteome Research, 14*(5), 1993-2001. doi:10.1021/pr501138h

Wei, R., Wang, J., Su, M., Jia, E., Chen, S., Chen, T., & Ni, Y. (2018). Missing Value Imputation Approach for Mass Spectrometry-based Metabolomics Data. *Scientific Reports, 8*(663). doi:10.1038/s41598-017-19120-0

Wishart, D. S., Feunang, Y. D., Marco, A., Guo, A. C., Liang, K., Vazquez-Fresno, R., . . . Scalbert, A. (2018, January). HMDB 4.0 - The Human Metabolome Database for 2018. *Nucleic Acid Res., 4*(46(D1)), D608-617. doi:10.1093/nar/gkx1089

Xia, J., & Wishart, D. S. (2011). Web-based inference of biological patterns, functions and pathways from metabolomic data using MetaboAnalyst. *Nature Protocols, 6*(6), 743-760. doi:10.1038/nprot.2011.319

Xia, J., Psychogios, N., Young, N., & Wishart, D. S. (2009). MetaboAnalyst: a web server for metabolomic data analysis and interpretation. *Nucleic Acids Research, 37*. doi:10.1093/nar/gkp356

Yang, J., Zhao, X., Lu, X., Lin, X., & Xu, G. (2015, February). A data preprocessing strategy for metabolomics to reduce the mask effect in data analysis. *Frontiers in Molecular Biosciences*. doi:10.3389/fmolb.2015.00004

**Tables**

Table 1: Sample Size Distribution of 2805 untargeted LC-ms data sets from Metabolon, Inc.

| Number of Samples | Percent of Total |
| --- | --- |
| 1 – 50 | 66.50% |
| 51 - 100 | 15.60% |
| 101 - 200 | 9.10% |
| 201 - 500 | 3.70% |
| 500 - 1000 | 2.20% |
| 1001+ | 2.00% |

Table 2: Distribution of Missing Values for the data set (1051 metabolites total).

| PERCENT PRESENT | NUMBER OF METABOLITES | PERCENT OF METABOLITES |
| --- | --- | --- |
| 100 | 675 | 64.2 |
| 90-99 | 165 | 15.7 |
| 80-89 | 53 | 5.0 |
| 70-79 | 24 | 2.3 |
| 60-69 | 29 | 2.8 |
| 50-59 | 22 | 2.1 |
| 40-49 | 20 | 1.9 |
| 30-39 | 15 | 1.4 |
| 20-29 | 14 | 1.3 |
| 10-19 | 14 | 1.3 |
| <10 | 20 | 1.9 |

Table 3: Percent of non-missing samples by FED/FASTED status, top metabolites by difference

| METABOLITE | NOT_FASTED | FASTED |
| --- | --- | --- |
| ferulic acid 4-sulfate | 96% | 22% |
| caffeic acid sulfate | 85% | 17% |
| 1-oleoyl-GPG (18:1)* | 88% | 26% |
| 4-vinylguaiacol sulfate | 88% | 26% |
| vanillic alcohol sulfate | 73% | 13% |
| 3-hydroxy-2-methylpyridine sulfate | 88% | 30% |
| trans-2-hexenoylglycine | 35% | 91% |
| 3-hydroxypyridine glucuronide | 69% | 13% |
| feruloylquinate (4) | 69% | 13% |
| 3-methyl catechol sulfate (2) | 73% | 17% |
| 3-acetylphenol sulfate | 77% | 22% |
| palmitoyl-myristoyl-glycerol (16:0/14:0) [1]* | 85% | 30% |
| N-palmitoylserine | 88% | 35% |
| 1-carboxyethyltyrosine | 58% | 4% |
| 2-furoylcarnitine | 65% | 13% |
| vanillic acid glycine | 100% | 48% |
| vanillate | 58% | 9% |
| maltol sulfate | 65% | 17% |
| 1-carboxyethylisoleucine | 85% | 39% |
| 1-palmitoyl-GPG (16:0)* | 81% | 39% |
| 5-hydroxy-2-methylpyridine sulfate | 81% | 39% |
| N-oleoyltaurine | 31% | 70% |
| dihydroferulate | 69% | 30% |
| 5alpha-androstan-3beta,17alpha-diol disulfate | 62% | 100% |
| diacylglycerol (12:0/18:1, 14:0/16:1, 16:0/14:1) [1]* | 81% | 43% |
| 4-acetylcatechol sulfate (2) | 50% | 13% |
| hexanoylglycine | 15% | 52% |
| alpha-hydroxycaproate | 54% | 17% |
| 4-acetylcatechol sulfate (1) | 92% | 57% |
| palmitoyl-myristoyl-glycerol (16:0/14:0) [2] | 92% | 57% |
| taurocholate | 96% | 61% |

Table 4: Distribution of Peak Areas for the Recovered Peaks (n=2351)

| PEAK AREA | PERCENT |
| --- | --- |
| < 10,000 | 17.8 |
| 10,000 - 20,000 | 17.2 |
| 20,000 - 50,000 | 34 |
| 50,000 - 100,000 | 15.1 |
| 100,000 - 250,000 | 10.8 |
| 250,000 - 500,000 | 3.1 |
| 500,000 - 1,000,000 | 1.1 |
| 1,000,000 - 10,000,000 | 0.7 |

Table 5: Distribution of Peaks Areas for the Recovered Peaks (5135 peaks) and no peaks

| RANGE | PERCENT |
| --- | --- |
| No Peak | 54.2 |
| Below the minimum | 36 |
| Between the min and Q1 | 8.1 |
| Between Q1 and the median | 1.3 |
| Between the median and Q3 | 0.2 |
| Between Q3 and the maximum | 0.2 |
| Above the maximum | 0 |

Table 6: Distribution of Peaks Areas for the Recovered Peaks (2351 peaks)

| RANGE | PERCENT |
| --- | --- |
| Below the minimum | 78.7 |
| Between the min and Q1 | 17.7 |
| Between Q1 and the median | 2.8 |
| Between the median and Q3 | 0.5 |
| Between Q3 and the maximum | 0.3 |
| Above the maximum | 0 |

Table 7: Median Absolute Difference of log10 p-values from the Recovered (162 metabolites)

| METHOD | MEDIAN | Q1 | Q3 |
| --- | --- | --- | --- |
| NONE | 0.227 | 0.110 | 0.433 |
| MIN | 0.092 | 0.043 | 0.195 |
| PMIN | 0.086 | 0.041 | 0.202 |
| HMIN | 0.094 | 0.032 | 0.194 |
| THRESH | 0.216 | 0.104 | 0.503 |
| RF | 0.185 | 0.092 | 0.303 |
| PLS | 0.185 | 0.100 | 0.320 |
| MICE(pmm,10) | 0.134 | 0.059 | 0.282 |
| MICE(norm,10) | 0.111 | 0.043 | 0.254 |
| KNN(obs,5,eucl,wtd,all(10)) | 0.138 | 0.079 | 0.294 |
| KNN(var,10,corr,wtd) | 0.141 | 0.068 | 0.235 |
| KNN(var,10,eucl,wtd) | 0.156 | 0.083 | 0.301 |
| KNN(var,10,corr,wtd,T) | 0.139 | 0.061 | 0.230 |
| KNN(var,10,eucl,wtd,T) | 0.143 | 0.076 | 0.268 |

**Figures**

Figure 1: Missingness patterns for theanine metabolites

Figure 2: Missingess Patterns for 1-carboxyethyl amino acids

Figure 3: Recovered Peaks vs. Original Peaks

z-scores computed based on the log-transform of the peaks originally found by the software


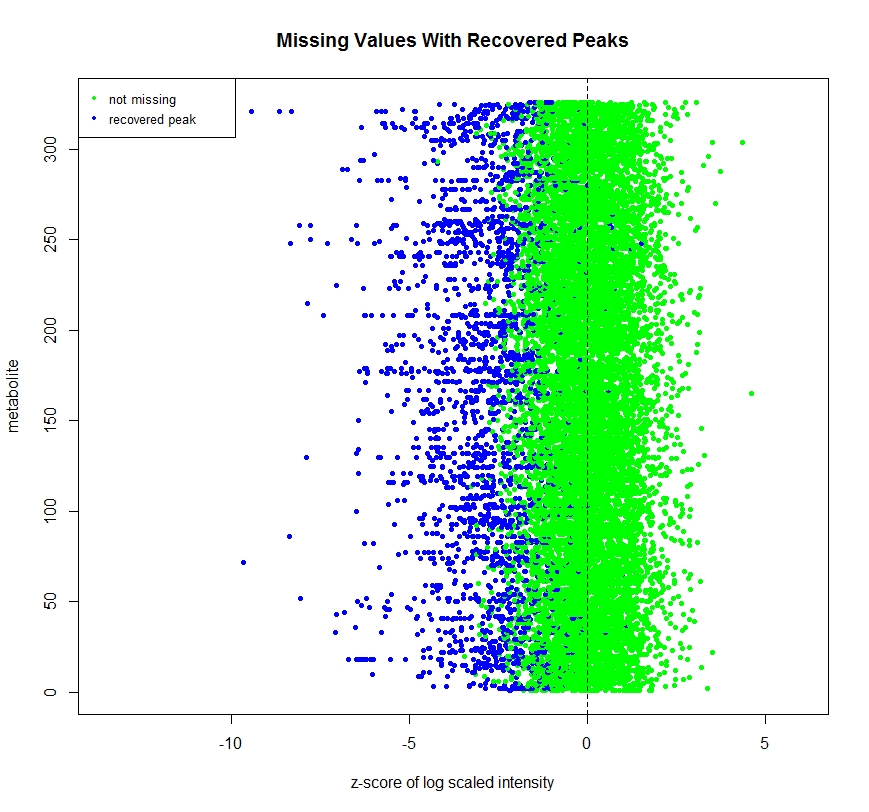


Figure 4: Type I Error, two-sample t-test, simulation study

Figure 5: Power for two-sample t-test, simulated data, mu=0.2, sd=0.3

Figure 6: Comparison of KNN imputations, data set simulations


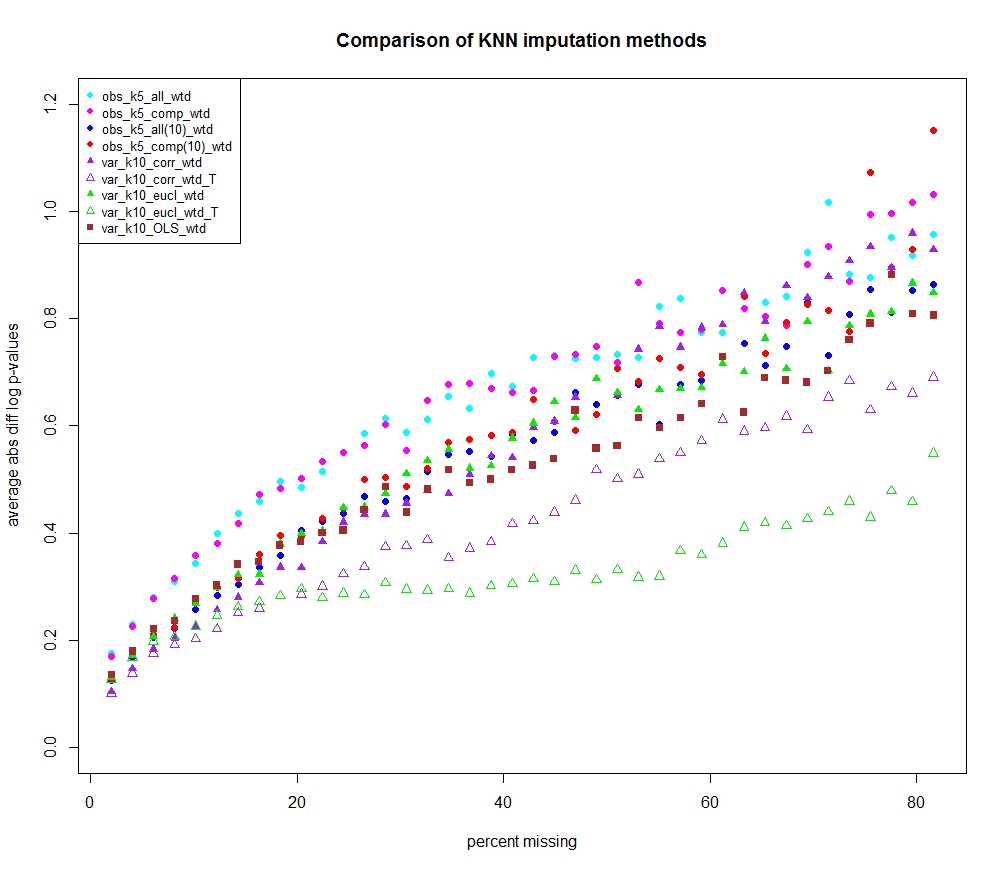


Figure 7: Mean Abs Error Data Simulation


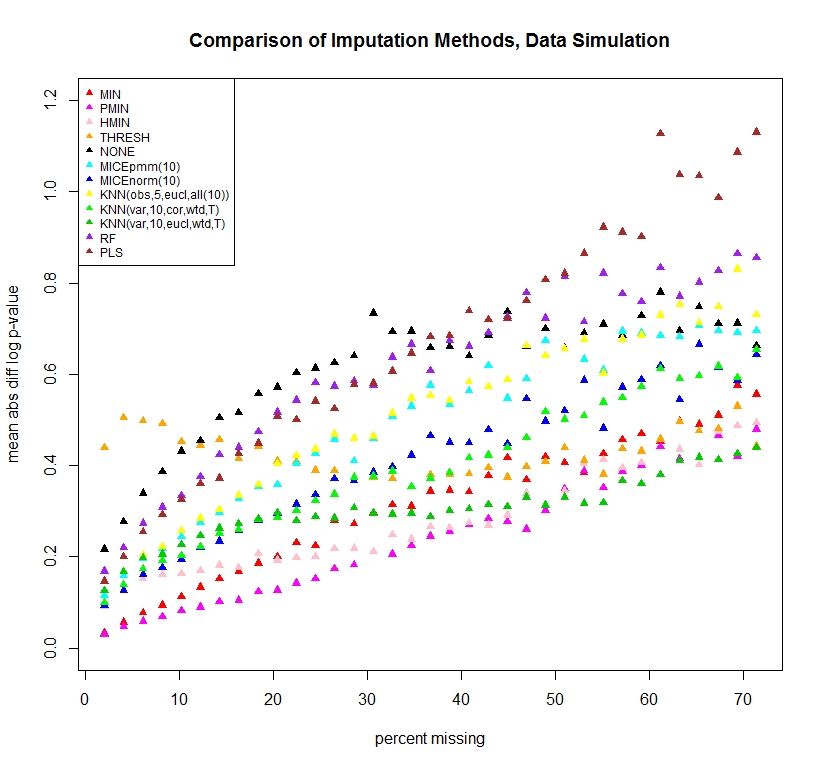


**Appendix:** Power, two-sample t-tests

Table A01: n1=n2=5, Type I error (nominal level 0.05)

| TYPE | PMISS | POW |
| --- | --- | --- |
| halfmin | 0.1 | 0.018 |
| min | 0.1 | 0.045 |
| none | 0.1 | 0.043 |
| prop | 0.1 | 0.045 |
| triangular | 0.1 | 0.027 |
| uniform | 0.1 | 0.021 |
| halfmin | 0.2 | 0.032 |
| min | 0.2 | 0.038 |
| none | 0.2 | 0.044 |
| prop | 0.2 | 0.050 |
| triangular | 0.2 | 0.027 |
| uniform | 0.2 | 0.017 |
| halfmin | 0.3 | 0.057 |
| min | 0.3 | 0.032 |
| none | 0.3 | 0.049 |
| prop | 0.3 | 0.061 |
| triangular | 0.3 | 0.032 |
| uniform | 0.3 | 0.015 |
| halfmin | 0.4 | 0.040 |
| min | 0.4 | 0.023 |
| none | 0.4 | 0.038 |
| prop | 0.4 | 0.043 |
| triangular | 0.4 | 0.031 |
| uniform | 0.4 | 0.022 |
| halfmin | 0.5 | 0.054 |
| min | 0.5 | 0.011 |
| none | 0.5 | 0.030 |
| prop | 0.5 | 0.056 |
| triangular | 0.5 | 0.030 |
| uniform | 0.5 | 0.023 |
| halfmin | 0.6 | 0.046 |
| min | 0.6 | 0.000 |
| none | 0.6 | 0.013 |
| prop | 0.6 | 0.047 |
| triangular | 0.6 | 0.029 |
| uniform | 0.6 | 0.023 |

Table A02: n1=n2=10, Type I error (nominal level 0.05)

| TYPE | PMISS | POW |  | TYPE | PMISS | POW |
| --- | --- | --- | --- | --- | --- | --- |
| halfmin | 0.05 | 0.038 |  | halfmin | 0.4 | 0.051 |
| min | 0.05 | 0.048 |  | min | 0.4 | 0.044 |
| none | 0.05 | 0.048 |  | none | 0.4 | 0.046 |
| prop | 0.05 | 0.051 |  | prop | 0.4 | 0.052 |
| triangular | 0.05 | 0.041 |  | triangular | 0.4 | 0.041 |
| uniform | 0.05 | 0.032 |  | uniform | 0.4 | 0.031 |
| halfmin | 0.1 | 0.044 |  | halfmin | 0.5 | 0.045 |
| min | 0.1 | 0.049 |  | min | 0.5 | 0.036 |
| none | 0.1 | 0.047 |  | none | 0.5 | 0.043 |
| prop | 0.1 | 0.053 |  | prop | 0.5 | 0.047 |
| triangular | 0.1 | 0.040 |  | triangular | 0.5 | 0.041 |
| uniform | 0.1 | 0.024 |  | uniform | 0.5 | 0.033 |
| halfmin | 0.2 | 0.046 |  | halfmin | 0.6 | 0.039 |
| min | 0.2 | 0.046 |  | min | 0.6 | 0.029 |
| none | 0.2 | 0.046 |  | none | 0.6 | 0.044 |
| prop | 0.2 | 0.050 |  | prop | 0.6 | 0.037 |
| triangular | 0.2 | 0.037 |  | triangular | 0.6 | 0.041 |
| uniform | 0.2 | 0.026 |  | uniform | 0.6 | 0.035 |
| halfmin | 0.3 | 0.052 |  | halfmin | 0.7 | 0.043 |
| min | 0.3 | 0.046 |  | min | 0.7 | 0.015 |
| none | 0.3 | 0.044 |  | none | 0.7 | 0.035 |
| prop | 0.3 | 0.049 |  | prop | 0.7 | 0.041 |
| triangular | 0.3 | 0.041 |  | triangular | 0.7 | 0.037 |
| uniform | 0.3 | 0.032 |  | uniform | 0.7 | 0.042 |

Table A03: n1=n2=20, Type I error (nominal level = 0.05)

| TYPE | PMISS | POW |  | TYPE | PMISS | POW |
| --- | --- | --- | --- | --- | --- | --- |
| halfmin | 0.05 | 0.049 |  | halfmin | 0.5 | 0.050 |
| min | 0.05 | 0.048 |  | min | 0.5 | 0.051 |
| none | 0.05 | 0.047 |  | none | 0.5 | 0.047 |
| prop | 0.05 | 0.052 |  | prop | 0.5 | 0.050 |
| triangular | 0.05 | 0.043 |  | triangular | 0.5 | 0.046 |
| uniform | 0.05 | 0.037 |  | uniform | 0.5 | 0.043 |
| halfmin | 0.1 | 0.046 |  | halfmin | 0.6 | 0.051 |
| min | 0.1 | 0.049 |  | min | 0.6 | 0.042 |
| none | 0.1 | 0.050 |  | none | 0.6 | 0.048 |
| prop | 0.1 | 0.050 |  | prop | 0.6 | 0.048 |
| triangular | 0.1 | 0.044 |  | triangular | 0.6 | 0.046 |
| uniform | 0.1 | 0.034 |  | uniform | 0.6 | 0.044 |
| halfmin | 0.2 | 0.053 |  | halfmin | 0.7 | 0.055 |
| min | 0.2 | 0.051 |  | min | 0.7 | 0.039 |
| none | 0.2 | 0.052 |  | none | 0.7 | 0.046 |
| prop | 0.2 | 0.051 |  | prop | 0.7 | 0.065 |
| triangular | 0.2 | 0.044 |  | triangular | 0.7 | 0.048 |
| uniform | 0.2 | 0.039 |  | uniform | 0.7 | 0.045 |
| halfmin | 0.3 | 0.052 |  | halfmin | 0.8 | 0.041 |
| min | 0.3 | 0.049 |  | min | 0.8 | 0.027 |
| none | 0.3 | 0.045 |  | none | 0.8 | 0.044 |
| prop | 0.3 | 0.052 |  | prop | 0.8 | 0.045 |
| triangular | 0.3 | 0.045 |  | triangular | 0.8 | 0.046 |
| uniform | 0.3 | 0.042 |  | uniform | 0.8 | 0.042 |
| halfmin | 0.4 | 0.055 |  |  |  |  |
| min | 0.4 | 0.051 |  |  |  |  |
| none | 0.4 | 0.047 |  |  |  |  |
| prop | 0.4 | 0.052 |  |  |  |  |
| triangular | 0.4 | 0.046 |  |  |  |  |
| uniform | 0.4 | 0.045 |  |  |  |  |

Table A04: n1=n2=30, Type I error (nominal level = 0.05)

| TYPE | PMISS | POW |  | TYPE | PMISS | POW |
| --- | --- | --- | --- | --- | --- | --- |
| halfmin | 0.05 | 0.044 |  | halfmin | 0.5 | 0.051 |
| min | 0.05 | 0.047 |  | min | 0.5 | 0.050 |
| none | 0.05 | 0.048 |  | none | 0.5 | 0.049 |
| prop | 0.05 | 0.051 |  | prop | 0.5 | 0.054 |
| triangular | 0.05 | 0.044 |  | triangular | 0.5 | 0.050 |
| uniform | 0.05 | 0.040 |  | uniform | 0.5 | 0.047 |
| halfmin | 0.1 | 0.048 |  | halfmin | 0.6 | 0.052 |
| min | 0.1 | 0.049 |  | min | 0.6 | 0.047 |
| none | 0.1 | 0.047 |  | none | 0.6 | 0.045 |
| prop | 0.1 | 0.052 |  | prop | 0.6 | 0.056 |
| triangular | 0.1 | 0.046 |  | triangular | 0.6 | 0.046 |
| uniform | 0.1 | 0.038 |  | uniform | 0.6 | 0.051 |
| halfmin | 0.2 | 0.045 |  | halfmin | 0.7 | 0.048 |
| min | 0.2 | 0.049 |  | min | 0.7 | 0.041 |
| none | 0.2 | 0.047 |  | none | 0.7 | 0.045 |
| prop | 0.2 | 0.046 |  | prop | 0.7 | 0.052 |
| triangular | 0.2 | 0.048 |  | triangular | 0.7 | 0.051 |
| uniform | 0.2 | 0.041 |  | uniform | 0.7 | 0.047 |
| halfmin | 0.3 | 0.047 |  | halfmin | 0.8 | 0.050 |
| min | 0.3 | 0.051 |  | min | 0.8 | 0.038 |
| none | 0.3 | 0.051 |  | none | 0.8 | 0.047 |
| prop | 0.3 | 0.050 |  | prop | 0.8 | 0.047 |
| triangular | 0.3 | 0.045 |  | triangular | 0.8 | 0.046 |
| uniform | 0.3 | 0.047 |  | uniform | 0.8 | 0.046 |
| halfmin | 0.4 | 0.050 |  |  |  |  |
| min | 0.4 | 0.049 |  |  |  |  |
| none | 0.4 | 0.048 |  |  |  |  |
| prop | 0.4 | 0.052 |  |  |  |  |
| triangular | 0.4 | 0.048 |  |  |  |  |
| uniform | 0.4 | 0.043 |  |  |  |  |

Table A05: n1=n2=50, Type I error (nominal level 0.05)

| TYPE | PMISS | POW |  | TYPE | PMISS | POW |
| --- | --- | --- | --- | --- | --- | --- |
| halfmin | 0.05 | 0.047 |  | halfmin | 0.5 | 0.051 |
| min | 0.05 | 0.050 |  | min | 0.5 | 0.046 |
| none | 0.05 | 0.051 |  | none | 0.5 | 0.046 |
| prop | 0.05 | 0.053 |  | prop | 0.5 | 0.049 |
| triangular | 0.05 | 0.046 |  | triangular | 0.5 | 0.049 |
| uniform | 0.05 | 0.045 |  | uniform | 0.5 | 0.048 |
| halfmin | 0.1 | 0.048 |  | halfmin | 0.6 | 0.049 |
| min | 0.1 | 0.054 |  | min | 0.6 | 0.049 |
| none | 0.1 | 0.047 |  | none | 0.6 | 0.048 |
| prop | 0.1 | 0.051 |  | prop | 0.6 | 0.051 |
| triangular | 0.1 | 0.052 |  | triangular | 0.6 | 0.050 |
| uniform | 0.1 | 0.046 |  | uniform | 0.6 | 0.050 |
| halfmin | 0.2 | 0.048 |  | halfmin | 0.7 | 0.047 |
| min | 0.2 | 0.050 |  | min | 0.7 | 0.044 |
| none | 0.2 | 0.050 |  | none | 0.7 | 0.050 |
| prop | 0.2 | 0.049 |  | prop | 0.7 | 0.049 |
| triangular | 0.2 | 0.048 |  | triangular | 0.7 | 0.051 |
| uniform | 0.2 | 0.050 |  | uniform | 0.7 | 0.047 |
| halfmin | 0.3 | 0.054 |  | halfmin | 0.8 | 0.054 |
| min | 0.3 | 0.050 |  | min | 0.8 | 0.046 |
| none | 0.3 | 0.051 |  | none | 0.8 | 0.047 |
| prop | 0.3 | 0.049 |  | prop | 0.8 | 0.057 |
| triangular | 0.3 | 0.047 |  | triangular | 0.8 | 0.046 |
| uniform | 0.3 | 0.050 |  | uniform | 0.8 | 0.048 |
| halfmin | 0.4 | 0.052 |  |  |  |  |
| min | 0.4 | 0.049 |  |  |  |  |
| none | 0.4 | 0.047 |  |  |  |  |
| prop | 0.4 | 0.049 |  |  |  |  |
| triangular | 0.4 | 0.048 |  |  |  |  |
| uniform | 0.4 | 0.046 |  |  |  |  |

Table A06: n1=n2=100, Type I error (nominal level 0.05)

| TYPE | PMISS | POW |  | TYPE | PMISS | POW |
| --- | --- | --- | --- | --- | --- | --- |
| halfmin | 0.05 | 0.045 |  | halfmin | 0.5 | 0.051 |
| min | 0.05 | 0.049 |  | min | 0.5 | 0.050 |
| none | 0.05 | 0.049 |  | none | 0.5 | 0.047 |
| prop | 0.05 | 0.048 |  | prop | 0.5 | 0.049 |
| triangular | 0.05 | 0.047 |  | triangular | 0.5 | 0.046 |
| uniform | 0.05 | 0.046 |  | uniform | 0.5 | 0.047 |
| halfmin | 0.1 | 0.049 |  | halfmin | 0.6 | 0.051 |
| min | 0.1 | 0.051 |  | min | 0.6 | 0.048 |
| none | 0.1 | 0.051 |  | none | 0.6 | 0.049 |
| prop | 0.1 | 0.053 |  | prop | 0.6 | 0.050 |
| triangular | 0.1 | 0.052 |  | triangular | 0.6 | 0.049 |
| uniform | 0.1 | 0.047 |  | uniform | 0.6 | 0.049 |
| halfmin | 0.2 | 0.047 |  | halfmin | 0.7 | 0.046 |
| min | 0.2 | 0.051 |  | min | 0.7 | 0.046 |
| none | 0.2 | 0.049 |  | none | 0.7 | 0.045 |
| prop | 0.2 | 0.052 |  | prop | 0.7 | 0.051 |
| triangular | 0.2 | 0.048 |  | triangular | 0.7 | 0.051 |
| uniform | 0.2 | 0.045 |  | uniform | 0.7 | 0.050 |
| halfmin | 0.3 | 0.052 |  | halfmin | 0.8 | 0.053 |
| min | 0.3 | 0.047 |  | min | 0.8 | 0.050 |
| none | 0.3 | 0.053 |  | none | 0.8 | 0.046 |
| prop | 0.3 | 0.053 |  | prop | 0.8 | 0.049 |
| triangular | 0.3 | 0.047 |  | triangular | 0.8 | 0.047 |
| uniform | 0.3 | 0.051 |  | uniform | 0.8 | 0.050 |
| halfmin | 0.4 | 0.052 |  |  |  |  |
| min | 0.4 | 0.049 |  |  |  |  |
| none | 0.4 | 0.054 |  |  |  |  |
| prop | 0.4 | 0.047 |  |  |  |  |
| triangular | 0.4 | 0.049 |  |  |  |  |
| uniform | 0.4 | 0.051 |  |  |  |  |

Table A07: n1=n2=5, mu=0.15, sigma=0.3 (true power approx. 0.093)

| TYPE | PMISS | POW |
| --- | --- | --- |
| halfmin | 0.1 | 0.052 |
| min | 0.1 | 0.099 |
| none | 0.1 | 0.076 |
| prop | 0.1 | 0.100 |
| triangular | 0.1 | 0.065 |
| uniform | 0.1 | 0.048 |
| halfmin | 0.2 | 0.073 |
| min | 0.2 | 0.086 |
| none | 0.2 | 0.075 |
| prop | 0.2 | 0.109 |
| triangular | 0.2 | 0.067 |
| uniform | 0.2 | 0.038 |
| halfmin | 0.3 | 0.121 |
| min | 0.3 | 0.070 |
| none | 0.3 | 0.063 |
| prop | 0.3 | 0.119 |
| triangular | 0.3 | 0.068 |
| uniform | 0.3 | 0.043 |
| halfmin | 0.4 | 0.083 |
| min | 0.4 | 0.051 |
| none | 0.4 | 0.044 |
| prop | 0.4 | 0.084 |
| triangular | 0.4 | 0.067 |
| uniform | 0.4 | 0.045 |
| halfmin | 0.5 | 0.108 |
| min | 0.5 | 0.028 |
| none | 0.5 | 0.033 |
| prop | 0.5 | 0.104 |
| triangular | 0.5 | 0.057 |
| uniform | 0.5 | 0.048 |
| halfmin | 0.6 | 0.091 |
| min | 0.6 | 0.000 |
| none | 0.6 | 0.013 |
| prop | 0.6 | 0.089 |
| triangular | 0.6 | 0.048 |
| uniform | 0.6 | 0.043 |

Table A08: n1=n2=10, mu=0.15, sigma=0.3 (true power approx. 0.188)

| TYPE | PMISS | POW |  | TYPE | PMISS | POW |
| --- | --- | --- | --- | --- | --- | --- |
| halfmin | 0.05 | 0.152 |  | halfmin | 0.4 | 0.168 |
| min | 0.05 | 0.179 |  | min | 0.4 | 0.143 |
| none | 0.05 | 0.157 |  | none | 0.4 | 0.086 |
| prop | 0.05 | 0.183 |  | prop | 0.4 | 0.174 |
| triangular | 0.05 | 0.150 |  | triangular | 0.4 | 0.118 |
| uniform | 0.05 | 0.132 |  | uniform | 0.4 | 0.094 |
| halfmin | 0.1 | 0.158 |  | halfmin | 0.5 | 0.155 |
| min | 0.1 | 0.176 |  | min | 0.5 | 0.119 |
| none | 0.1 | 0.135 |  | none | 0.5 | 0.080 |
| prop | 0.1 | 0.185 |  | prop | 0.5 | 0.147 |
| triangular | 0.1 | 0.146 |  | triangular | 0.5 | 0.098 |
| uniform | 0.1 | 0.115 |  | uniform | 0.5 | 0.092 |
| halfmin | 0.2 | 0.156 |  | halfmin | 0.6 | 0.124 |
| min | 0.2 | 0.171 |  | min | 0.6 | 0.088 |
| none | 0.2 | 0.116 |  | none | 0.6 | 0.062 |
| prop | 0.2 | 0.183 |  | prop | 0.6 | 0.121 |
| triangular | 0.2 | 0.140 |  | triangular | 0.6 | 0.082 |
| uniform | 0.2 | 0.104 |  | uniform | 0.6 | 0.079 |
| halfmin | 0.3 | 0.171 |  | halfmin | 0.7 | 0.124 |
| min | 0.3 | 0.160 |  | min | 0.7 | 0.043 |
| none | 0.3 | 0.095 |  | none | 0.7 | 0.036 |
| prop | 0.3 | 0.179 |  | prop | 0.7 | 0.111 |
| triangular | 0.3 | 0.134 |  | triangular | 0.7 | 0.062 |
| uniform | 0.3 | 0.098 |  | uniform | 0.7 | 0.063 |

Table A09: n1=n2=20, mu=0.15, sigma=0.3 (true power approx. 0.338)

| TYPE | PMISS | POW |  | TYPE | PMISS | POW |
| --- | --- | --- | --- | --- | --- | --- |
| halfmin | 0.05 | 0.317 |  | halfmin | 0.5 | 0.275 |
| min | 0.05 | 0.337 |  | min | 0.5 | 0.245 |
| none | 0.05 | 0.279 |  | none | 0.5 | 0.116 |
| prop | 0.05 | 0.340 |  | prop | 0.5 | 0.283 |
| triangular | 0.05 | 0.301 |  | triangular | 0.5 | 0.180 |
| uniform | 0.05 | 0.255 |  | uniform | 0.5 | 0.160 |
| halfmin | 0.1 | 0.299 |  | halfmin | 0.6 | 0.264 |
| min | 0.1 | 0.327 |  | min | 0.6 | 0.211 |
| none | 0.1 | 0.239 |  | none | 0.6 | 0.093 |
| prop | 0.1 | 0.333 |  | prop | 0.6 | 0.259 |
| triangular | 0.1 | 0.288 |  | triangular | 0.6 | 0.141 |
| uniform | 0.1 | 0.243 |  | uniform | 0.6 | 0.129 |
| halfmin | 0.2 | 0.297 |  | halfmin | 0.7 | 0.244 |
| min | 0.2 | 0.315 |  | min | 0.7 | 0.169 |
| none | 0.2 | 0.185 |  | none | 0.7 | 0.085 |
| prop | 0.2 | 0.333 |  | prop | 0.7 | 0.265 |
| triangular | 0.2 | 0.251 |  | triangular | 0.7 | 0.105 |
| uniform | 0.2 | 0.214 |  | uniform | 0.7 | 0.101 |
| halfmin | 0.3 | 0.296 |  | halfmin | 0.8 | 0.173 |
| min | 0.3 | 0.298 |  | min | 0.8 | 0.103 |
| none | 0.3 | 0.154 |  | none | 0.8 | 0.052 |
| prop | 0.3 | 0.316 |  | prop | 0.8 | 0.180 |
| triangular | 0.3 | 0.245 |  | triangular | 0.8 | 0.069 |
| uniform | 0.3 | 0.202 |  | uniform | 0.8 | 0.075 |
| halfmin | 0.4 | 0.288 |  |  |  |  |
| min | 0.4 | 0.279 |  |  |  |  |
| none | 0.4 | 0.124 |  |  |  |  |
| prop | 0.4 | 0.293 |  |  |  |  |
| triangular | 0.4 | 0.211 |  |  |  |  |
| uniform | 0.4 | 0.172 |  |  |  |  |

Table A10: n1=n2=30, mu=0.15, sigma=0.3 (true power approx. 0.484)

| TYPE | PMISS | POW |  | TYPE | PMISS | POW |
| --- | --- | --- | --- | --- | --- | --- |
| halfmin | 0.05 | 0.439 |  | halfmin | 0.5 | 0.385 |
| min | 0.05 | 0.467 |  | min | 0.5 | 0.361 |
| none | 0.05 | 0.405 |  | none | 0.5 | 0.133 |
| prop | 0.05 | 0.470 |  | prop | 0.5 | 0.391 |
| triangular | 0.05 | 0.429 |  | triangular | 0.5 | 0.243 |
| uniform | 0.05 | 0.392 |  | uniform | 0.5 | 0.211 |
| halfmin | 0.1 | 0.440 |  | halfmin | 0.6 | 0.351 |
| min | 0.1 | 0.459 |  | min | 0.6 | 0.325 |
| none | 0.1 | 0.341 |  | none | 0.6 | 0.116 |
| prop | 0.1 | 0.467 |  | prop | 0.6 | 0.373 |
| triangular | 0.1 | 0.408 |  | triangular | 0.6 | 0.191 |
| uniform | 0.1 | 0.345 |  | uniform | 0.6 | 0.173 |
| halfmin | 0.2 | 0.428 |  | halfmin | 0.7 | 0.314 |
| min | 0.2 | 0.452 |  | min | 0.7 | 0.268 |
| none | 0.2 | 0.260 |  | none | 0.7 | 0.099 |
| prop | 0.2 | 0.461 |  | prop | 0.7 | 0.303 |
| triangular | 0.2 | 0.377 |  | triangular | 0.7 | 0.136 |
| uniform | 0.2 | 0.301 |  | uniform | 0.7 | 0.131 |
| halfmin | 0.3 | 0.431 |  | halfmin | 0.8 | 0.279 |
| min | 0.3 | 0.423 |  | min | 0.8 | 0.195 |
| none | 0.3 | 0.211 |  | none | 0.8 | 0.084 |
| prop | 0.3 | 0.452 |  | prop | 0.8 | 0.257 |
| triangular | 0.3 | 0.337 |  | triangular | 0.8 | 0.084 |
| uniform | 0.3 | 0.265 |  | uniform | 0.8 | 0.096 |
| halfmin | 0.4 | 0.407 |  |  |  |  |
| min | 0.4 | 0.399 |  |  |  |  |
| none | 0.4 | 0.168 |  |  |  |  |
| prop | 0.4 | 0.427 |  |  |  |  |
| triangular | 0.4 | 0.302 |  |  |  |  |
| uniform | 0.4 | 0.243 |  |  |  |  |

Table A11: n1=n2=50, mu=0.15, sigma=0.3 (true power approx. 0.693)

| TYPE | PMISS | POW |  | TYPE | PMISS | POW |
| --- | --- | --- | --- | --- | --- | --- |
| halfmin | 0.05 | 0.659 |  | halfmin | 0.5 | 0.583 |
| min | 0.05 | 0.692 |  | min | 0.5 | 0.559 |
| none | 0.05 | 0.589 |  | none | 0.5 | 0.190 |
| prop | 0.05 | 0.695 |  | prop | 0.5 | 0.591 |
| triangular | 0.05 | 0.638 |  | triangular | 0.5 | 0.367 |
| uniform | 0.05 | 0.570 |  | uniform | 0.5 | 0.317 |
| halfmin | 0.1 | 0.648 |  | halfmin | 0.6 | 0.548 |
| min | 0.1 | 0.685 |  | min | 0.6 | 0.503 |
| none | 0.1 | 0.524 |  | none | 0.6 | 0.151 |
| prop | 0.1 | 0.682 |  | prop | 0.6 | 0.543 |
| triangular | 0.1 | 0.596 |  | triangular | 0.6 | 0.288 |
| uniform | 0.1 | 0.507 |  | uniform | 0.6 | 0.254 |
| halfmin | 0.2 | 0.631 |  | halfmin | 0.7 | 0.515 |
| min | 0.2 | 0.667 |  | min | 0.7 | 0.432 |
| none | 0.2 | 0.399 |  | none | 0.7 | 0.121 |
| prop | 0.2 | 0.677 |  | prop | 0.7 | 0.483 |
| triangular | 0.2 | 0.554 |  | triangular | 0.7 | 0.201 |
| uniform | 0.2 | 0.446 |  | uniform | 0.7 | 0.189 |
| halfmin | 0.3 | 0.628 |  | halfmin | 0.8 | 0.434 |
| min | 0.3 | 0.631 |  | min | 0.8 | 0.332 |
| none | 0.3 | 0.315 |  | none | 0.8 | 0.105 |
| prop | 0.3 | 0.648 |  | prop | 0.8 | 0.454 |
| triangular | 0.3 | 0.508 |  | triangular | 0.8 | 0.110 |
| uniform | 0.3 | 0.405 |  | uniform | 0.8 | 0.132 |
| halfmin | 0.4 | 0.619 |  |  |  |  |
| min | 0.4 | 0.595 |  |  |  |  |
| none | 0.4 | 0.249 |  |  |  |  |
| prop | 0.4 | 0.628 |  |  |  |  |
| triangular | 0.4 | 0.447 |  |  |  |  |
| uniform | 0.4 | 0.357 |  |  |  |  |

Table A12: n1=n2=100, mu=0.15, sigma=0.3 (true power approx. 0.941)

| TYPE | PMISS | POW |  | TYPE | PMISS | POW |
| --- | --- | --- | --- | --- | --- | --- |
| halfmin | 0.05 | 0.919 |  | halfmin | 0.5 | 0.874 |
| min | 0.05 | 0.933 |  | min | 0.5 | 0.850 |
| none | 0.05 | 0.879 |  | none | 0.5 | 0.325 |
| prop | 0.05 | 0.940 |  | prop | 0.5 | 0.872 |
| triangular | 0.05 | 0.897 |  | triangular | 0.5 | 0.625 |
| uniform | 0.05 | 0.834 |  | uniform | 0.5 | 0.538 |
| halfmin | 0.1 | 0.908 |  | halfmin | 0.6 | 0.849 |
| min | 0.1 | 0.938 |  | min | 0.6 | 0.802 |
| none | 0.1 | 0.814 |  | none | 0.6 | 0.236 |
| prop | 0.1 | 0.940 |  | prop | 0.6 | 0.839 |
| triangular | 0.1 | 0.877 |  | triangular | 0.6 | 0.502 |
| uniform | 0.1 | 0.774 |  | uniform | 0.6 | 0.450 |
| halfmin | 0.2 | 0.908 |  | halfmin | 0.7 | 0.798 |
| min | 0.2 | 0.923 |  | min | 0.7 | 0.734 |
| none | 0.2 | 0.677 |  | none | 0.7 | 0.179 |
| prop | 0.2 | 0.933 |  | prop | 0.7 | 0.780 |
| triangular | 0.2 | 0.835 |  | triangular | 0.7 | 0.351 |
| uniform | 0.2 | 0.718 |  | uniform | 0.7 | 0.327 |
| halfmin | 0.3 | 0.892 |  | halfmin | 0.8 | 0.712 |
| min | 0.3 | 0.899 |  | min | 0.8 | 0.614 |
| none | 0.3 | 0.536 |  | none | 0.8 | 0.130 |
| prop | 0.3 | 0.916 |  | prop | 0.8 | 0.684 |
| triangular | 0.3 | 0.796 |  | triangular | 0.8 | 0.188 |
| uniform | 0.3 | 0.665 |  | uniform | 0.8 | 0.219 |
| halfmin | 0.4 | 0.893 |  |  |  |  |
| min | 0.4 | 0.882 |  |  |  |  |
| none | 0.4 | 0.425 |  |  |  |  |
| prop | 0.4 | 0.903 |  |  |  |  |
| triangular | 0.4 | 0.720 |  |  |  |  |
| uniform | 0.4 | 0.619 |  |  |  |  |

Table A13: n1=n2=5, mu=0.2, sigma=0.3 (true power approx. 0.143)

| TYPE | PMISS | POW |
| --- | --- | --- |
| halfmin | 0.1 | 0.084 |
| min | 0.1 | 0.136 |
| none | 0.1 | 0.111 |
| prop | 0.1 | 0.149 |
| triangular | 0.1 | 0.096 |
| uniform | 0.1 | 0.074 |
| halfmin | 0.2 | 0.113 |
| min | 0.2 | 0.123 |
| none | 0.2 | 0.092 |
| prop | 0.2 | 0.153 |
| triangular | 0.2 | 0.105 |
| uniform | 0.2 | 0.057 |
| halfmin | 0.3 | 0.166 |
| min | 0.3 | 0.099 |
| none | 0.3 | 0.080 |
| prop | 0.3 | 0.165 |
| triangular | 0.3 | 0.097 |
| uniform | 0.3 | 0.061 |
| halfmin | 0.4 | 0.118 |
| min | 0.4 | 0.070 |
| none | 0.4 | 0.057 |
| prop | 0.4 | 0.120 |
| triangular | 0.4 | 0.091 |
| uniform | 0.4 | 0.069 |
| halfmin | 0.5 | 0.146 |
| min | 0.5 | 0.036 |
| none | 0.5 | 0.029 |
| prop | 0.5 | 0.145 |
| triangular | 0.5 | 0.073 |
| uniform | 0.5 | 0.063 |
| halfmin | 0.6 | 0.130 |
| min | 0.6 | 0.000 |
| none | 0.6 | 0.009 |
| prop | 0.6 | 0.129 |
| triangular | 0.6 | 0.053 |
| uniform | 0.6 | 0.055 |

Table A14: n1=n2=10, mu=0.2, sigma=0.3 (true power approx. 0.289)

| TYPE | PMISS | POW |  | TYPE | PMISS | POW |
| --- | --- | --- | --- | --- | --- | --- |
| halfmin | 0.05 | 0.250 |  | halfmin | 0.4 | 0.263 |
| min | 0.05 | 0.287 |  | min | 0.4 | 0.220 |
| none | 0.05 | 0.240 |  | none | 0.4 | 0.114 |
| prop | 0.05 | 0.282 |  | prop | 0.4 | 0.265 |
| triangular | 0.05 | 0.239 |  | triangular | 0.4 | 0.185 |
| uniform | 0.05 | 0.209 |  | uniform | 0.4 | 0.153 |
| halfmin | 0.1 | 0.255 |  | halfmin | 0.5 | 0.244 |
| min | 0.1 | 0.291 |  | min | 0.5 | 0.188 |
| none | 0.1 | 0.212 |  | none | 0.5 | 0.093 |
| prop | 0.1 | 0.291 |  | prop | 0.5 | 0.240 |
| triangular | 0.1 | 0.234 |  | triangular | 0.5 | 0.152 |
| uniform | 0.1 | 0.181 |  | uniform | 0.5 | 0.140 |
| halfmin | 0.2 | 0.252 |  | halfmin | 0.6 | 0.198 |
| min | 0.2 | 0.271 |  | min | 0.6 | 0.137 |
| none | 0.2 | 0.170 |  | none | 0.6 | 0.069 |
| prop | 0.2 | 0.286 |  | prop | 0.6 | 0.184 |
| triangular | 0.2 | 0.231 |  | triangular | 0.6 | 0.116 |
| uniform | 0.2 | 0.171 |  | uniform | 0.6 | 0.111 |
| halfmin | 0.3 | 0.272 |  | halfmin | 0.7 | 0.187 |
| min | 0.3 | 0.250 |  | min | 0.7 | 0.067 |
| none | 0.3 | 0.138 |  | none | 0.7 | 0.036 |
| prop | 0.3 | 0.277 |  | prop | 0.7 | 0.156 |
| triangular | 0.3 | 0.203 |  | triangular | 0.7 | 0.078 |
| uniform | 0.3 | 0.169 |  | uniform | 0.7 | 0.091 |

Table A15: n1=n2=20, mu=0.2, sigma=0.3 (true power approx. 0.537)

| TYPE | PMISS | POW |  | TYPE | PMISS | POW |
| --- | --- | --- | --- | --- | --- | --- |
| halfmin | 0.05 | 0.502 |  | halfmin | 0.5 | 0.451 |
| min | 0.05 | 0.529 |  | min | 0.5 | 0.405 |
| none | 0.05 | 0.451 |  | none | 0.5 | 0.147 |
| prop | 0.05 | 0.529 |  | prop | 0.5 | 0.441 |
| triangular | 0.05 | 0.483 |  | triangular | 0.5 | 0.284 |
| uniform | 0.05 | 0.437 |  | uniform | 0.5 | 0.242 |
| halfmin | 0.1 | 0.488 |  | halfmin | 0.6 | 0.422 |
| min | 0.1 | 0.537 |  | min | 0.6 | 0.349 |
| none | 0.1 | 0.395 |  | none | 0.6 | 0.133 |
| prop | 0.1 | 0.531 |  | prop | 0.6 | 0.425 |
| triangular | 0.1 | 0.466 |  | triangular | 0.6 | 0.209 |
| uniform | 0.1 | 0.403 |  | uniform | 0.6 | 0.183 |
| halfmin | 0.2 | 0.479 |  | halfmin | 0.7 | 0.389 |
| min | 0.2 | 0.510 |  | min | 0.7 | 0.284 |
| none | 0.2 | 0.306 |  | none | 0.7 | 0.115 |
| prop | 0.2 | 0.520 |  | prop | 0.7 | 0.421 |
| triangular | 0.2 | 0.432 |  | triangular | 0.7 | 0.140 |
| uniform | 0.2 | 0.345 |  | uniform | 0.7 | 0.147 |
| halfmin | 0.3 | 0.475 |  | halfmin | 0.8 | 0.279 |
| min | 0.3 | 0.482 |  | min | 0.8 | 0.166 |
| none | 0.3 | 0.235 |  | none | 0.8 | 0.056 |
| prop | 0.3 | 0.503 |  | prop | 0.8 | 0.280 |
| triangular | 0.3 | 0.396 |  | triangular | 0.8 | 0.085 |
| uniform | 0.3 | 0.313 |  | uniform | 0.8 | 0.095 |
| halfmin | 0.4 | 0.465 |  |  |  |  |
| min | 0.4 | 0.446 |  |  |  |  |
| none | 0.4 | 0.192 |  |  |  |  |
| prop | 0.4 | 0.475 |  |  |  |  |
| triangular | 0.4 | 0.342 |  |  |  |  |
| uniform | 0.4 | 0.284 |  |  |  |  |

Table A16: n1=n2=30, mu=0.2, sigma=0.3 (true power approx. 0.726)

| TYPE | PMISS | POW |  | TYPE | PMISS | POW |
| --- | --- | --- | --- | --- | --- | --- |
| halfmin | 0.05 | 0.676 |  | halfmin | 0.5 | 0.618 |
| min | 0.05 | 0.716 |  | min | 0.5 | 0.566 |
| none | 0.05 | 0.631 |  | none | 0.5 | 0.191 |
| prop | 0.05 | 0.720 |  | prop | 0.5 | 0.607 |
| triangular | 0.05 | 0.663 |  | triangular | 0.5 | 0.381 |
| uniform | 0.05 | 0.602 |  | uniform | 0.5 | 0.334 |
| halfmin | 0.1 | 0.659 |  | halfmin | 0.6 | 0.572 |
| min | 0.1 | 0.715 |  | min | 0.6 | 0.514 |
| none | 0.1 | 0.546 |  | none | 0.6 | 0.160 |
| prop | 0.1 | 0.722 |  | prop | 0.6 | 0.559 |
| triangular | 0.1 | 0.643 |  | triangular | 0.6 | 0.291 |
| uniform | 0.1 | 0.548 |  | uniform | 0.6 | 0.276 |
| halfmin | 0.2 | 0.650 |  | halfmin | 0.7 | 0.511 |
| min | 0.2 | 0.678 |  | min | 0.7 | 0.430 |
| none | 0.2 | 0.422 |  | none | 0.7 | 0.130 |
| prop | 0.2 | 0.703 |  | prop | 0.7 | 0.490 |
| triangular | 0.2 | 0.583 |  | triangular | 0.7 | 0.202 |
| uniform | 0.2 | 0.484 |  | uniform | 0.7 | 0.202 |
| halfmin | 0.3 | 0.652 |  | halfmin | 0.8 | 0.434 |
| min | 0.3 | 0.653 |  | min | 0.8 | 0.316 |
| none | 0.3 | 0.332 |  | none | 0.8 | 0.107 |
| prop | 0.3 | 0.682 |  | prop | 0.8 | 0.409 |
| triangular | 0.3 | 0.541 |  | triangular | 0.8 | 0.105 |
| uniform | 0.3 | 0.427 |  | uniform | 0.8 | 0.130 |
| halfmin | 0.4 | 0.641 |  |  |  |  |
| min | 0.4 | 0.624 |  |  |  |  |
| none | 0.4 | 0.256 |  |  |  |  |
| prop | 0.4 | 0.643 |  |  |  |  |
| triangular | 0.4 | 0.473 |  |  |  |  |
| uniform | 0.4 | 0.393 |  |  |  |  |

Table A17: n1=n2=50, mu=0.2, sigma=0.3 (true power approx. 0.913)

| TYPE | PMISS | POW |  | TYPE | PMISS | POW |
| --- | --- | --- | --- | --- | --- | --- |
| halfmin | 0.05 | 0.881 |  | halfmin | 0.5 | 0.832 |
| min | 0.05 | 0.905 |  | min | 0.5 | 0.805 |
| none | 0.05 | 0.834 |  | none | 0.5 | 0.291 |
| prop | 0.05 | 0.912 |  | prop | 0.5 | 0.825 |
| triangular | 0.05 | 0.864 |  | triangular | 0.5 | 0.575 |
| uniform | 0.05 | 0.797 |  | uniform | 0.5 | 0.497 |
| halfmin | 0.1 | 0.873 |  | halfmin | 0.6 | 0.799 |
| min | 0.1 | 0.902 |  | min | 0.6 | 0.751 |
| none | 0.1 | 0.764 |  | none | 0.6 | 0.225 |
| prop | 0.1 | 0.908 |  | prop | 0.6 | 0.794 |
| triangular | 0.1 | 0.832 |  | triangular | 0.6 | 0.442 |
| uniform | 0.1 | 0.746 |  | uniform | 0.6 | 0.397 |
| halfmin | 0.2 | 0.869 |  | halfmin | 0.7 | 0.740 |
| min | 0.2 | 0.883 |  | min | 0.7 | 0.666 |
| none | 0.2 | 0.630 |  | none | 0.7 | 0.176 |
| prop | 0.2 | 0.897 |  | prop | 0.7 | 0.722 |
| triangular | 0.2 | 0.794 |  | triangular | 0.7 | 0.300 |
| uniform | 0.2 | 0.676 |  | uniform | 0.7 | 0.306 |
| halfmin | 0.3 | 0.856 |  | halfmin | 0.8 | 0.661 |
| min | 0.3 | 0.867 |  | min | 0.8 | 0.531 |
| none | 0.3 | 0.487 |  | none | 0.8 | 0.145 |
| prop | 0.3 | 0.880 |  | prop | 0.8 | 0.678 |
| triangular | 0.3 | 0.750 |  | triangular | 0.8 | 0.161 |
| uniform | 0.3 | 0.629 |  | uniform | 0.8 | 0.192 |
| halfmin | 0.4 | 0.840 |  |  |  |  |
| min | 0.4 | 0.838 |  |  |  |  |
| none | 0.4 | 0.391 |  |  |  |  |
| prop | 0.4 | 0.865 |  |  |  |  |
| triangular | 0.4 | 0.681 |  |  |  |  |
| uniform | 0.4 | 0.569 |  |  |  |  |

Table A18: n1=n2=100, mu=0.2, sigma=0.3 (true power approx. 0.997)

| TYPE | PMISS | POW |  | TYPE | PMISS | POW |
| --- | --- | --- | --- | --- | --- | --- |
| halfmin | 0.05 | 0.994 |  | halfmin | 0.5 | 0.985 |
| min | 0.05 | 0.997 |  | min | 0.5 | 0.981 |
| none | 0.05 | 0.986 |  | none | 0.5 | 0.510 |
| prop | 0.05 | 0.997 |  | prop | 0.5 | 0.985 |
| triangular | 0.05 | 0.991 |  | triangular | 0.5 | 0.865 |
| uniform | 0.05 | 0.962 |  | uniform | 0.5 | 0.785 |
| halfmin | 0.1 | 0.992 |  | halfmin | 0.6 | 0.977 |
| min | 0.1 | 0.995 |  | min | 0.6 | 0.964 |
| none | 0.1 | 0.965 |  | none | 0.6 | 0.380 |
| prop | 0.1 | 0.997 |  | prop | 0.6 | 0.976 |
| triangular | 0.1 | 0.985 |  | triangular | 0.6 | 0.727 |
| uniform | 0.1 | 0.943 |  | uniform | 0.6 | 0.676 |
| halfmin | 0.2 | 0.991 |  | halfmin | 0.7 | 0.963 |
| min | 0.2 | 0.996 |  | min | 0.7 | 0.928 |
| none | 0.2 | 0.900 |  | none | 0.7 | 0.269 |
| prop | 0.2 | 0.994 |  | prop | 0.7 | 0.959 |
| triangular | 0.2 | 0.972 |  | triangular | 0.7 | 0.522 |
| uniform | 0.2 | 0.913 |  | uniform | 0.7 | 0.513 |
| halfmin | 0.3 | 0.992 |  | halfmin | 0.8 | 0.916 |
| min | 0.3 | 0.993 |  | min | 0.8 | 0.847 |
| none | 0.3 | 0.788 |  | none | 0.8 | 0.190 |
| prop | 0.3 | 0.995 |  | prop | 0.8 | 0.907 |
| triangular | 0.3 | 0.955 |  | triangular | 0.8 | 0.266 |
| uniform | 0.3 | 0.890 |  | uniform | 0.8 | 0.328 |
| halfmin | 0.4 | 0.988 |  |  |  |  |
| min | 0.4 | 0.987 |  |  |  |  |
| none | 0.4 | 0.650 |  |  |  |  |
| prop | 0.4 | 0.991 |  |  |  |  |
| triangular | 0.4 | 0.923 |  |  |  |  |
| uniform | 0.4 | 0.846 |  |  |  |  |

Table A19: n1=n2=5, mu=0.25, sigma=0.3 (true power approx. 0.202)

| TYPE | PMISS | POW |
| --- | --- | --- |
| halfmin | 0.1 | 0.132 |
| min | 0.1 | 0.195 |
| none | 0.1 | 0.146 |
| prop | 0.1 | 0.205 |
| triangular | 0.1 | 0.141 |
| uniform | 0.1 | 0.106 |
| halfmin | 0.2 | 0.159 |
| min | 0.2 | 0.174 |
| none | 0.2 | 0.117 |
| prop | 0.2 | 0.213 |
| triangular | 0.2 | 0.139 |
| uniform | 0.2 | 0.081 |
| halfmin | 0.3 | 0.235 |
| min | 0.3 | 0.138 |
| none | 0.3 | 0.096 |
| prop | 0.3 | 0.220 |
| triangular | 0.3 | 0.140 |
| uniform | 0.3 | 0.090 |
| halfmin | 0.4 | 0.164 |
| min | 0.4 | 0.100 |
| none | 0.4 | 0.065 |
| prop | 0.4 | 0.164 |
| triangular | 0.4 | 0.127 |
| uniform | 0.4 | 0.099 |
| halfmin | 0.5 | 0.195 |
| min | 0.5 | 0.052 |
| none | 0.5 | 0.030 |
| prop | 0.5 | 0.190 |
| triangular | 0.5 | 0.103 |
| uniform | 0.5 | 0.085 |
| halfmin | 0.6 | 0.173 |
| min | 0.6 | 0.000 |
| none | 0.6 | 0.009 |
| prop | 0.6 | 0.167 |
| triangular | 0.6 | 0.068 |
| uniform | 0.6 | 0.074 |

Table A20: n1=n2=10, mu=0.25, sigma=0.3 (true power approx. 0.289)

| TYPE | PMISS | POW |  | TYPE | PMISS | POW |
| --- | --- | --- | --- | --- | --- | --- |
| halfmin | 0.05 | 0.378 |  | halfmin | 0.4 | 0.381 |
| min | 0.05 | 0.414 |  | min | 0.4 | 0.323 |
| none | 0.05 | 0.360 |  | none | 0.4 | 0.153 |
| prop | 0.05 | 0.417 |  | prop | 0.4 | 0.380 |
| triangular | 0.05 | 0.366 |  | triangular | 0.4 | 0.274 |
| uniform | 0.05 | 0.323 |  | uniform | 0.4 | 0.215 |
| halfmin | 0.1 | 0.383 |  | halfmin | 0.5 | 0.340 |
| min | 0.1 | 0.410 |  | min | 0.5 | 0.267 |
| none | 0.1 | 0.305 |  | none | 0.5 | 0.130 |
| prop | 0.1 | 0.408 |  | prop | 0.5 | 0.344 |
| triangular | 0.1 | 0.354 |  | triangular | 0.5 | 0.222 |
| uniform | 0.1 | 0.282 |  | uniform | 0.5 | 0.191 |
| halfmin | 0.2 | 0.368 |  | halfmin | 0.6 | 0.293 |
| min | 0.2 | 0.401 |  | min | 0.6 | 0.199 |
| none | 0.2 | 0.233 |  | none | 0.6 | 0.082 |
| prop | 0.2 | 0.411 |  | prop | 0.6 | 0.266 |
| triangular | 0.2 | 0.342 |  | triangular | 0.6 | 0.158 |
| uniform | 0.2 | 0.260 |  | uniform | 0.6 | 0.154 |
| halfmin | 0.3 | 0.382 |  | halfmin | 0.7 | 0.259 |
| min | 0.3 | 0.363 |  | min | 0.7 | 0.094 |
| none | 0.3 | 0.189 |  | none | 0.7 | 0.027 |
| prop | 0.3 | 0.396 |  | prop | 0.7 | 0.241 |
| triangular | 0.3 | 0.317 |  | triangular | 0.7 | 0.100 |
| uniform | 0.3 | 0.251 |  | uniform | 0.7 | 0.116 |

Table A21: n1=n2=20, mu=0.25, sigma=0.3 (true power approx. 0.724)

| TYPE | PMISS | POW |  | TYPE | PMISS | POW |
| --- | --- | --- | --- | --- | --- | --- |
| halfmin | 0.05 | 0.690 |  | halfmin | 0.5 | 0.625 |
| min | 0.05 | 0.724 |  | min | 0.5 | 0.570 |
| none | 0.05 | 0.634 |  | none | 0.5 | 0.216 |
| prop | 0.05 | 0.724 |  | prop | 0.5 | 0.628 |
| triangular | 0.05 | 0.672 |  | triangular | 0.5 | 0.401 |
| uniform | 0.05 | 0.611 |  | uniform | 0.5 | 0.352 |
| halfmin | 0.1 | 0.671 |  | halfmin | 0.6 | 0.595 |
| min | 0.1 | 0.716 |  | min | 0.6 | 0.499 |
| none | 0.1 | 0.567 |  | none | 0.6 | 0.175 |
| prop | 0.1 | 0.720 |  | prop | 0.6 | 0.586 |
| triangular | 0.1 | 0.661 |  | triangular | 0.6 | 0.295 |
| uniform | 0.1 | 0.573 |  | uniform | 0.6 | 0.273 |
| halfmin | 0.2 | 0.659 |  | halfmin | 0.7 | 0.539 |
| min | 0.2 | 0.701 |  | min | 0.7 | 0.403 |
| none | 0.2 | 0.435 |  | none | 0.7 | 0.141 |
| prop | 0.2 | 0.714 |  | prop | 0.7 | 0.568 |
| triangular | 0.2 | 0.612 |  | triangular | 0.7 | 0.184 |
| uniform | 0.2 | 0.511 |  | uniform | 0.7 | 0.206 |
| halfmin | 0.3 | 0.660 |  | halfmin | 0.8 | 0.404 |
| min | 0.3 | 0.661 |  | min | 0.8 | 0.251 |
| none | 0.3 | 0.347 |  | none | 0.8 | 0.056 |
| prop | 0.3 | 0.692 |  | prop | 0.8 | 0.412 |
| triangular | 0.3 | 0.557 |  | triangular | 0.8 | 0.103 |
| uniform | 0.3 | 0.459 |  | uniform | 0.8 | 0.126 |
| halfmin | 0.4 | 0.643 |  |  |  |  |
| min | 0.4 | 0.622 |  |  |  |  |
| none | 0.4 | 0.269 |  |  |  |  |
| prop | 0.4 | 0.661 |  |  |  |  |
| triangular | 0.4 | 0.486 |  |  |  |  |
| uniform | 0.4 | 0.413 |  |  |  |  |

Table A22: n1=n2=30, mu=0.25, sigma=0.3 (true power approx. 0.884)

| TYPE | PMISS | POW |  | TYPE | PMISS | POW |
| --- | --- | --- | --- | --- | --- | --- |
| halfmin | 0.05 | 0.861 |  | halfmin | 0.5 | 0.793 |
| min | 0.05 | 0.882 |  | min | 0.5 | 0.769 |
| none | 0.05 | 0.805 |  | none | 0.5 | 0.280 |
| prop | 0.05 | 0.886 |  | prop | 0.5 | 0.803 |
| triangular | 0.05 | 0.834 |  | triangular | 0.5 | 0.552 |
| uniform | 0.05 | 0.797 |  | uniform | 0.5 | 0.474 |
| halfmin | 0.1 | 0.850 |  | halfmin | 0.6 | 0.769 |
| min | 0.1 | 0.886 |  | min | 0.6 | 0.700 |
| none | 0.1 | 0.743 |  | none | 0.6 | 0.222 |
| prop | 0.1 | 0.881 |  | prop | 0.6 | 0.762 |
| triangular | 0.1 | 0.821 |  | triangular | 0.6 | 0.421 |
| uniform | 0.1 | 0.737 |  | uniform | 0.6 | 0.378 |
| halfmin | 0.2 | 0.833 |  | halfmin | 0.7 | 0.695 |
| min | 0.2 | 0.861 |  | min | 0.7 | 0.611 |
| none | 0.2 | 0.599 |  | none | 0.7 | 0.181 |
| prop | 0.2 | 0.868 |  | prop | 0.7 | 0.668 |
| triangular | 0.2 | 0.769 |  | triangular | 0.7 | 0.268 |
| uniform | 0.2 | 0.656 |  | uniform | 0.7 | 0.283 |
| halfmin | 0.3 | 0.834 |  | halfmin | 0.8 | 0.603 |
| min | 0.3 | 0.837 |  | min | 0.8 | 0.464 |
| none | 0.3 | 0.483 |  | none | 0.8 | 0.125 |
| prop | 0.3 | 0.856 |  | prop | 0.8 | 0.584 |
| triangular | 0.3 | 0.710 |  | triangular | 0.8 | 0.137 |
| uniform | 0.3 | 0.609 |  | uniform | 0.8 | 0.175 |
| halfmin | 0.4 | 0.822 |  |  |  |  |
| min | 0.4 | 0.815 |  |  |  |  |
| none | 0.4 | 0.368 |  |  |  |  |
| prop | 0.4 | 0.836 |  |  |  |  |
| triangular | 0.4 | 0.656 |  |  |  |  |
| uniform | 0.4 | 0.552 |  |  |  |  |

Table A23: n1=n2=50, mu=0.25, sigma=0.3 (true power approx. 0.985)

| TYPE | PMISS | POW |  | TYPE | PMISS | POW |
| --- | --- | --- | --- | --- | --- | --- |
| halfmin | 0.05 | 0.977 |  | halfmin | 0.5 | 0.954 |
| min | 0.05 | 0.984 |  | min | 0.5 | 0.934 |
| none | 0.05 | 0.960 |  | none | 0.5 | 0.416 |
| prop | 0.05 | 0.984 |  | prop | 0.5 | 0.954 |
| triangular | 0.05 | 0.965 |  | triangular | 0.5 | 0.765 |
| uniform | 0.05 | 0.927 |  | uniform | 0.5 | 0.672 |
| halfmin | 0.1 | 0.972 |  | halfmin | 0.6 | 0.936 |
| min | 0.1 | 0.982 |  | min | 0.6 | 0.908 |
| none | 0.1 | 0.921 |  | none | 0.6 | 0.312 |
| prop | 0.1 | 0.984 |  | prop | 0.6 | 0.930 |
| triangular | 0.1 | 0.954 |  | triangular | 0.6 | 0.599 |
| uniform | 0.1 | 0.893 |  | uniform | 0.6 | 0.573 |
| halfmin | 0.2 | 0.968 |  | halfmin | 0.7 | 0.908 |
| min | 0.2 | 0.976 |  | min | 0.7 | 0.846 |
| none | 0.2 | 0.821 |  | none | 0.7 | 0.233 |
| prop | 0.2 | 0.977 |  | prop | 0.7 | 0.893 |
| triangular | 0.2 | 0.931 |  | triangular | 0.7 | 0.402 |
| uniform | 0.2 | 0.847 |  | uniform | 0.7 | 0.430 |
| halfmin | 0.3 | 0.969 |  | halfmin | 0.8 | 0.835 |
| min | 0.3 | 0.968 |  | min | 0.8 | 0.726 |
| none | 0.3 | 0.680 |  | none | 0.8 | 0.186 |
| prop | 0.3 | 0.976 |  | prop | 0.8 | 0.846 |
| triangular | 0.3 | 0.897 |  | triangular | 0.8 | 0.190 |
| uniform | 0.3 | 0.806 |  | uniform | 0.8 | 0.257 |
| halfmin | 0.4 | 0.960 |  |  |  |  |
| min | 0.4 | 0.959 |  |  |  |  |
| none | 0.4 | 0.534 |  |  |  |  |
| prop | 0.4 | 0.966 |  |  |  |  |
| triangular | 0.4 | 0.853 |  |  |  |  |
| uniform | 0.4 | 0.750 |  |  |  |  |

Table A24: n1=n2=100, mu=0.25, sigma=0.3 (true power approx. 1.000)

| TYPE | PMISS | POW |  | TYPE | PMISS | POW |
| --- | --- | --- | --- | --- | --- | --- |
| halfmin | 0.05 | 1.000 |  | halfmin | 0.5 | 0.999 |
| min | 0.05 | 1.000 |  | min | 0.5 | 0.999 |
| none | 0.05 | 0.999 |  | none | 0.5 | 0.677 |
| prop | 0.05 | 1.000 |  | prop | 0.5 | 0.999 |
| triangular | 0.05 | 1.000 |  | triangular | 0.5 | 0.959 |
| uniform | 0.05 | 0.996 |  | uniform | 0.5 | 0.922 |
| halfmin | 0.1 | 1.000 |  | halfmin | 0.6 | 0.999 |
| min | 0.1 | 1.000 |  | min | 0.6 | 0.997 |
| none | 0.1 | 0.998 |  | none | 0.6 | 0.508 |
| prop | 0.1 | 1.000 |  | prop | 0.6 | 0.998 |
| triangular | 0.1 | 0.999 |  | triangular | 0.6 | 0.883 |
| uniform | 0.1 | 0.993 |  | uniform | 0.6 | 0.854 |
| halfmin | 0.2 | 1.000 |  | halfmin | 0.7 | 0.998 |
| min | 0.2 | 1.000 |  | min | 0.7 | 0.988 |
| none | 0.2 | 0.981 |  | none | 0.7 | 0.353 |
| prop | 0.2 | 1.000 |  | prop | 0.7 | 0.997 |
| triangular | 0.2 | 0.998 |  | triangular | 0.7 | 0.685 |
| uniform | 0.2 | 0.986 |  | uniform | 0.7 | 0.701 |
| halfmin | 0.3 | 1.000 |  | halfmin | 0.8 | 0.987 |
| min | 0.3 | 0.999 |  | min | 0.8 | 0.956 |
| none | 0.3 | 0.930 |  | none | 0.8 | 0.248 |
| prop | 0.3 | 1.000 |  | prop | 0.8 | 0.982 |
| triangular | 0.3 | 0.995 |  | triangular | 0.8 | 0.362 |
| uniform | 0.3 | 0.975 |  | uniform | 0.8 | 0.465 |
| halfmin | 0.4 | 1.000 |  |  |  |  |
| min | 0.4 | 0.999 |  |  |  |  |
| none | 0.4 | 0.832 |  |  |  |  |
| prop | 0.4 | 1.000 |  |  |  |  |
| triangular | 0.4 | 0.985 |  |  |  |  |
| uniform | 0.4 | 0.956 |  |  |  |  |

Table A25: n1=n2=5, mu=0.3, sigma=0.3 (true power approx. 0.262)

| TYPE | PMISS | POW |
| --- | --- | --- |
| halfmin | 0.1 | 0.175 |
| min | 0.1 | 0.270 |
| none | 0.1 | 0.204 |
| prop | 0.1 | 0.265 |
| triangular | 0.1 | 0.198 |
| uniform | 0.1 | 0.148 |
| halfmin | 0.2 | 0.229 |
| min | 0.2 | 0.226 |
| none | 0.2 | 0.162 |
| prop | 0.2 | 0.293 |
| triangular | 0.2 | 0.196 |
| uniform | 0.2 | 0.116 |
| halfmin | 0.3 | 0.312 |
| min | 0.3 | 0.186 |
| none | 0.3 | 0.126 |
| prop | 0.3 | 0.299 |
| triangular | 0.3 | 0.194 |
| uniform | 0.3 | 0.123 |
| halfmin | 0.4 | 0.220 |
| min | 0.4 | 0.130 |
| none | 0.4 | 0.070 |
| prop | 0.4 | 0.220 |
| triangular | 0.4 | 0.170 |
| uniform | 0.4 | 0.131 |
| halfmin | 0.5 | 0.260 |
| min | 0.5 | 0.076 |
| none | 0.5 | 0.026 |
| prop | 0.5 | 0.262 |
| triangular | 0.5 | 0.131 |
| uniform | 0.5 | 0.114 |
| halfmin | 0.6 | 0.225 |
| min | 0.6 | 0.000 |
| none | 0.6 | 0.008 |
| prop | 0.6 | 0.236 |
| triangular | 0.6 | 0.080 |
| uniform | 0.6 | 0.089 |
|  |  |  |

Table A26: n1=n2=10, mu=0.3, sigma=0.3 (true power approx. 0.560)

| TYPE | PMISS | POW |  | TYPE | PMISS | POW |
| --- | --- | --- | --- | --- | --- | --- |
| halfmin | 0.05 | 0.521 |  | halfmin | 0.4 | 0.509 |
| min | 0.05 | 0.561 |  | min | 0.4 | 0.439 |
| none | 0.05 | 0.486 |  | none | 0.4 | 0.201 |
| prop | 0.05 | 0.560 |  | prop | 0.4 | 0.509 |
| triangular | 0.05 | 0.504 |  | triangular | 0.4 | 0.365 |
| uniform | 0.05 | 0.438 |  | uniform | 0.4 | 0.304 |
| halfmin | 0.1 | 0.503 |  | halfmin | 0.5 | 0.458 |
| min | 0.1 | 0.544 |  | min | 0.5 | 0.380 |
| none | 0.1 | 0.425 |  | none | 0.5 | 0.164 |
| prop | 0.1 | 0.556 |  | prop | 0.5 | 0.468 |
| triangular | 0.1 | 0.487 |  | triangular | 0.5 | 0.292 |
| uniform | 0.1 | 0.399 |  | uniform | 0.5 | 0.256 |
| halfmin | 0.2 | 0.489 |  | halfmin | 0.6 | 0.402 |
| min | 0.2 | 0.516 |  | min | 0.6 | 0.280 |
| none | 0.2 | 0.322 |  | none | 0.6 | 0.085 |
| prop | 0.2 | 0.541 |  | prop | 0.6 | 0.376 |
| triangular | 0.2 | 0.469 |  | triangular | 0.6 | 0.194 |
| uniform | 0.2 | 0.361 |  | uniform | 0.6 | 0.209 |
| halfmin | 0.3 | 0.501 |  | halfmin | 0.7 | 0.352 |
| min | 0.3 | 0.493 |  | min | 0.7 | 0.135 |
| none | 0.3 | 0.250 |  | none | 0.7 | 0.026 |
| prop | 0.3 | 0.521 |  | prop | 0.7 | 0.322 |
| triangular | 0.3 | 0.426 |  | triangular | 0.7 | 0.125 |
| uniform | 0.3 | 0.343 |  | uniform | 0.7 | 0.145 |

Table A27: n1=n2=20, mu=0.3, sigma=0.3 (true power approx. 0.867)

| TYPE | PMISS | POW |  | TYPE | PMISS | POW |
| --- | --- | --- | --- | --- | --- | --- |
| halfmin | 0.05 | 0.839 |  | min | 0.5 | 0.738 |
| min | 0.05 | 0.859 |  | none | 0.5 | 0.281 |
| none | 0.05 | 0.791 |  | prop | 0.5 | 0.779 |
| prop | 0.05 | 0.867 |  | triangular | 0.5 | 0.530 |
| triangular | 0.05 | 0.832 |  | uniform | 0.5 | 0.463 |
| uniform | 0.05 | 0.783 |  | halfmin | 0.6 | 0.745 |
| halfmin | 0.1 | 0.831 |  | min | 0.6 | 0.662 |
| min | 0.1 | 0.854 |  | none | 0.6 | 0.218 |
| none | 0.1 | 0.723 |  | prop | 0.6 | 0.746 |
| prop | 0.1 | 0.868 |  | triangular | 0.6 | 0.386 |
| triangular | 0.1 | 0.801 |  | uniform | 0.6 | 0.375 |
| uniform | 0.1 | 0.728 |  | halfmin | 0.7 | 0.691 |
| halfmin | 0.2 | 0.822 |  | min | 0.7 | 0.552 |
| min | 0.2 | 0.841 |  | none | 0.7 | 0.151 |
| none | 0.2 | 0.579 |  | prop | 0.7 | 0.713 |
| prop | 0.2 | 0.853 |  | triangular | 0.7 | 0.232 |
| triangular | 0.2 | 0.751 |  | uniform | 0.7 | 0.272 |
| uniform | 0.2 | 0.655 |  | halfmin | 0.8 | 0.542 |
| halfmin | 0.3 | 0.812 |  | min | 0.8 | 0.337 |
| min | 0.3 | 0.813 |  | none | 0.8 | 0.048 |
| none | 0.3 | 0.448 |  | prop | 0.8 | 0.536 |
| prop | 0.3 | 0.836 |  | triangular | 0.8 | 0.119 |
| triangular | 0.3 | 0.711 |  | uniform | 0.8 | 0.161 |
| uniform | 0.3 | 0.605 |  |  |  |  |
| halfmin | 0.4 | 0.798 |  |  |  |  |
| min | 0.4 | 0.780 |  |  |  |  |
| none | 0.4 | 0.353 |  |  |  |  |
| prop | 0.4 | 0.809 |  |  |  |  |
| triangular | 0.4 | 0.626 |  |  |  |  |
| uniform | 0.4 | 0.543 |  |  |  |  |
| halfmin | 0.5 | 0.784 |  |  |  |  |

Table A28: n1=n2=30, mu=0.3, sigma=0.3 (true power approx. 0.971)

| TYPE | PMISS | POW |  | TYPE | PMISS | POW |
| --- | --- | --- | --- | --- | --- | --- |
| halfmin | 0.05 | 0.955 |  | halfmin | 0.5 | 0.918 |
| min | 0.05 | 0.968 |  | min | 0.5 | 0.894 |
| none | 0.05 | 0.928 |  | none | 0.5 | 0.369 |
| prop | 0.05 | 0.966 |  | prop | 0.5 | 0.919 |
| triangular | 0.05 | 0.943 |  | triangular | 0.5 | 0.688 |
| uniform | 0.05 | 0.904 |  | uniform | 0.5 | 0.621 |
| halfmin | 0.1 | 0.946 |  | halfmin | 0.6 | 0.892 |
| min | 0.1 | 0.964 |  | min | 0.6 | 0.847 |
| none | 0.1 | 0.881 |  | none | 0.6 | 0.279 |
| prop | 0.1 | 0.966 |  | prop | 0.6 | 0.894 |
| triangular | 0.1 | 0.927 |  | triangular | 0.6 | 0.532 |
| uniform | 0.1 | 0.855 |  | uniform | 0.6 | 0.513 |
| halfmin | 0.2 | 0.942 |  | halfmin | 0.7 | 0.846 |
| min | 0.2 | 0.956 |  | min | 0.7 | 0.758 |
| none | 0.2 | 0.751 |  | none | 0.7 | 0.220 |
| prop | 0.2 | 0.963 |  | prop | 0.7 | 0.828 |
| triangular | 0.2 | 0.894 |  | triangular | 0.7 | 0.338 |
| uniform | 0.2 | 0.807 |  | uniform | 0.7 | 0.368 |
| halfmin | 0.3 | 0.938 |  | halfmin | 0.8 | 0.757 |
| min | 0.3 | 0.941 |  | min | 0.8 | 0.592 |
| none | 0.3 | 0.621 |  | none | 0.8 | 0.127 |
| prop | 0.3 | 0.952 |  | prop | 0.8 | 0.745 |
| triangular | 0.3 | 0.852 |  | triangular | 0.8 | 0.161 |
| uniform | 0.3 | 0.758 |  | uniform | 0.8 | 0.222 |
| halfmin | 0.4 | 0.933 |  |  |  |  |
| min | 0.4 | 0.926 |  |  |  |  |
| none | 0.4 | 0.488 |  |  |  |  |
| prop | 0.4 | 0.938 |  |  |  |  |
| triangular | 0.4 | 0.792 |  |  |  |  |
| uniform | 0.4 | 0.697 |  |  |  |  |

Table A29: n1=n2=50, mu=0.3, sigma=0.3 (true power approx. 0.999)

| TYPE | PMISS | POW |  | TYPE | PMISS | POW |
| --- | --- | --- | --- | --- | --- | --- |
| halfmin | 0.05 | 0.997 |  | halfmin | 0.5 | 0.990 |
| min | 0.05 | 0.999 |  | min | 0.5 | 0.989 |
| none | 0.05 | 0.995 |  | none | 0.5 | 0.538 |
| prop | 0.05 | 0.999 |  | prop | 0.5 | 0.993 |
| triangular | 0.05 | 0.994 |  | triangular | 0.5 | 0.882 |
| uniform | 0.05 | 0.979 |  | uniform | 0.5 | 0.815 |
| halfmin | 0.1 | 0.996 |  | halfmin | 0.6 | 0.988 |
| min | 0.1 | 0.998 |  | min | 0.6 | 0.972 |
| none | 0.1 | 0.980 |  | none | 0.6 | 0.392 |
| prop | 0.1 | 0.999 |  | prop | 0.6 | 0.987 |
| triangular | 0.1 | 0.990 |  | triangular | 0.6 | 0.748 |
| uniform | 0.1 | 0.964 |  | uniform | 0.6 | 0.717 |
| halfmin | 0.2 | 0.996 |  | halfmin | 0.7 | 0.975 |
| min | 0.2 | 0.997 |  | min | 0.7 | 0.947 |
| none | 0.2 | 0.931 |  | none | 0.7 | 0.288 |
| prop | 0.2 | 0.997 |  | prop | 0.7 | 0.968 |
| triangular | 0.2 | 0.984 |  | triangular | 0.7 | 0.520 |
| uniform | 0.2 | 0.944 |  | uniform | 0.7 | 0.560 |
| halfmin | 0.3 | 0.996 |  | halfmin | 0.8 | 0.941 |
| min | 0.3 | 0.996 |  | min | 0.8 | 0.858 |
| none | 0.3 | 0.835 |  | none | 0.8 | 0.213 |
| prop | 0.3 | 0.996 |  | prop | 0.8 | 0.946 |
| triangular | 0.3 | 0.972 |  | triangular | 0.8 | 0.245 |
| uniform | 0.3 | 0.923 |  | uniform | 0.8 | 0.328 |
| halfmin | 0.4 | 0.994 |  |  |  |  |
| min | 0.4 | 0.993 |  |  |  |  |
| none | 0.4 | 0.690 |  |  |  |  |
| prop | 0.4 | 0.994 |  |  |  |  |
| triangular | 0.4 | 0.943 |  |  |  |  |
| uniform | 0.4 | 0.879 |  |  |  |  |

Table A30: n1=n2=100, mu=0.3, sigma=0.3 (true power approx. 1.000)

| TYPE | PMISS | POW |  | TYPE | PMISS | POW |
| --- | --- | --- | --- | --- | --- | --- |
| halfmin | 0.05 | 1.000 |  | halfmin | 0.5 | 1.000 |
| min | 0.05 | 1.000 |  | min | 0.5 | 1.000 |
| none | 0.05 | 1.000 |  | none | 0.5 | 0.806 |
| prop | 0.05 | 1.000 |  | prop | 0.5 | 1.000 |
| triangular | 0.05 | 1.000 |  | triangular | 0.5 | 0.991 |
| uniform | 0.05 | 1.000 |  | uniform | 0.5 | 0.980 |
| halfmin | 0.1 | 1.000 |  | halfmin | 0.6 | 1.000 |
| min | 0.1 | 1.000 |  | min | 0.6 | 1.000 |
| none | 0.1 | 1.000 |  | none | 0.6 | 0.631 |
| prop | 0.1 | 1.000 |  | prop | 0.6 | 1.000 |
| triangular | 0.1 | 1.000 |  | triangular | 0.6 | 0.961 |
| uniform | 0.1 | 1.000 |  | uniform | 0.6 | 0.941 |
| halfmin | 0.2 | 1.000 |  | halfmin | 0.7 | 1.000 |
| min | 0.2 | 1.000 |  | min | 0.7 | 0.999 |
| none | 0.2 | 0.998 |  | none | 0.7 | 0.444 |
| prop | 0.2 | 1.000 |  | prop | 0.7 | 1.000 |
| triangular | 0.2 | 1.000 |  | triangular | 0.7 | 0.803 |
| uniform | 0.2 | 0.999 |  | uniform | 0.7 | 0.847 |
| halfmin | 0.3 | 1.000 |  | halfmin | 0.8 | 0.999 |
| min | 0.3 | 1.000 |  | min | 0.8 | 0.992 |
| none | 0.3 | 0.984 |  | none | 0.8 | 0.288 |
| prop | 0.3 | 1.000 |  | prop | 0.8 | 0.998 |
| triangular | 0.3 | 1.000 |  | triangular | 0.8 | 0.437 |
| uniform | 0.3 | 0.996 |  | uniform | 0.8 | 0.581 |
| halfmin | 0.4 | 1.000 |  |  |  |  |
| min | 0.4 | 1.000 |  |  |  |  |
| none | 0.4 | 0.930 |  |  |  |  |
| prop | 0.4 | 1.000 |  |  |  |  |
| triangular | 0.4 | 0.999 |  |  |  |  |
| uniform | 0.4 | 0.990 |  |  |  |  |
