# Supplementary material for: Untargeted analysis of the serum metabolome in cats with exocrine pancreatic insufficiency
Source: PLoS One. 2021 Sep 30;16(9):e0257856. doi: 10.1371/journal.pone.0257856 (PMC8483406; doi:10.1371/journal.pone.0257856)
Supplement: S4 File — (DOCX) [file pone.0257856.s004.docx]

**Supplementary Table 1:** Significantly Altered Lipid Metabolites

| **Biochemical** | **Super-Pathway** | **Sub-Pathway** | **log2FC** | **P-Value** | **FDR** |
| --- | --- | --- | --- | --- | --- |
| palmitoyl-oleoyl-glycerol (16:0/18:1) [2] | Lipid | Diacylglycerol | -4.238 | 3E-07 | 6.49E-05 |
| stearoyl-linoleoyl-glycerol (18:0/18:2) [2] | Lipid | Diacylglycerol | -3.137 | 0.0004526 | 0.0056855 |
| palmitoyl-linoleoyl-glycerol (16:0/18:2) [1] | Lipid | Diacylglycerol | -2.883 | 0.0006623 | 0.0071323 |
| palmitoyl-linoleoyl-glycerol (16:0/18:2) [2] | Lipid | Diacylglycerol | -2.836 | 0.0002924 | 0.0049987 |
| oleoyl-oleoyl-glycerol (18:1/18:1)  [1] | Lipid | Diacylglycerol | -2.732 | 0.0002283 | 0.0049987 |
| oleoyl-oleoyl-glycerol (18:1/18:1) [2] | Lipid | Diacylglycerol | -2.518 | 1.2E-06 | 0.0001129 |
| diacylglycerol (16:1/18:2 [2], 16:0/18:3 [1]) | Lipid | Diacylglycerol | -1.935 | 0.000742 | 0.0075584 |
| palmitoyl-oleoyl-glycerol (16:0/18:1) [1] | Lipid | Diacylglycerol | -1.687 | 0.0002802 | 0.0049987 |
| oleoyl-linoleoyl-glycerol (18:1/18:2) [2] | Lipid | Diacylglycerol | -1.4 | 0.0013618 | 0.0101091 |
| oleoyl-linoleoyl-glycerol (18:1/18:2) [1] | Lipid | Diacylglycerol | -1.372 | 0.0028242 | 0.0158867 |
| palmitoyl-arachidonoyl-glycerol (16:0/20:4) [2] | Lipid | Diacylglycerol | -1.196 | 0.0017561 | 0.0116115 |
| N-arachidonoyltaurine | Lipid | Endocannabinoid | 1.399 | 0.0102814 | 0.0345977 |
| N-linolenoyltaurine | Lipid | Endocannabinoid | 2.166 | 0.0191025 | 0.0489764 |
| propionylglycine | Lipid | Fatty Acid Metabolism | -1.536 | 0.0381581 | 0.0756917 |
| butyrylglycine | Lipid | Fatty Acid Metabolism | -0.817 | 0.0274448 | 0.0636363 |
| dihomo-linolenoylcarnitine (20:3n3 or 6) | Lipid | Fatty Acid Metabolism (Acyl Carnitine) | -1.775 | 0.0128817 | 0.0392005 |
| myristoylcarnitine (C14) | Lipid | Fatty Acid Metabolism (Acyl Carnitine) | 0.729 | 0.0444097 | 0.0816467 |
| 2-aminoheptanoate | Lipid | Fatty Acid, Amino | -1.447 | 0.0039049 | 0.0186292 |
| 17-methylstearate | Lipid | Fatty Acid, Branched | 0.947 | 0.0009902 | 0.0088382 |
| 15-methylpalmitate | Lipid | Fatty Acid, Branched | 1.5 | 0.0026573 | 0.0153708 |
| 3-methyladipate | Lipid | Fatty Acid, Dicarboxylate | -1.026 | 0.0048529 | 0.0213603 |
| 3-hydroxylaurate | Lipid | Fatty Acid, Monohydroxy | 0.71 | 0.0054803 | 0.0229498 |
| 3-hydroxydecanoate | Lipid | Fatty Acid, Monohydroxy | 0.786 | 0.0076946 | 0.0290002 |
| glycerophosphoglycerol | Lipid | Glycerolipid Metabolism | -2.281 | 0.0001436 | 0.0039818 |
| myo-inositol | Lipid | Inositol Metabolism | -1.176 | 0.0008588 | 0.0085175 |
| stearate (18:0) | Lipid | Long Chain Fatty Acid | 0.81 | 0.0292104 | 0.0655599 |
| palmitate (16:0) | Lipid | Long Chain Fatty Acid | 0.821 | 0.0428612 | 0.0796784 |
| myristate (14:0) | Lipid | Long Chain Fatty Acid | 0.948 | 0.0088876 | 0.0313051 |
| oleate/vaccenate (18:1) | Lipid | Long Chain Fatty Acid | 1.092 | 0.0187221 | 0.0486631 |
| eicosenoate (20:1) | Lipid | Long Chain Fatty Acid | 1.317 | 0.0362443 | 0.0746454 |
| margarate (17:0) | Lipid | Long Chain Fatty Acid | 1.528 | 0.0118335 | 0.0384476 |
| 10-nonadecenoate (19:1n9) | Lipid | Long Chain Fatty Acid | 1.647 | 0.0018827 | 0.0120265 |
| 10-heptadecenoate (17:1n7) | Lipid | Long Chain Fatty Acid | 1.852 | 0.0005413 | 0.0061823 |
| 1-linoleoyl-GPE (18:2) | Lipid | Lysophospholipid | -1.781 | 0.0004067 | 0.0056776 |
| 1-linoleoyl-GPS (18:2) | Lipid | Lysophospholipid | -1.702 | 0.0056378 | 0.02335 |
| 1-oleoyl-GPE (18:1) | Lipid | Lysophospholipid | -1.53 | 0.0002935 | 0.0049987 |
| 1-palmitoyl-GPG (16:0) | Lipid | Lysophospholipid | -1.27 | 0.0181745 | 0.0475679 |
| 1-arachidonoyl-GPE (20:4n6) | Lipid | Lysophospholipid | -0.967 | 0.0181076 | 0.0475679 |
| 1-stearoyl-GPG (18:0) | Lipid | Lysophospholipid | -0.886 | 0.0158428 | 0.0440985 |
| 1-oleoyl-GPG (18:1) | Lipid | Lysophospholipid | -0.873 | 0.0052875 | 0.0226453 |
| 1-stearoyl-GPE (18:0) | Lipid | Lysophospholipid | -0.808 | 0.0159129 | 0.0440985 |
| 1-palmitoyl-GPE (16:0) | Lipid | Lysophospholipid | -0.772 | 0.0125561 | 0.0391096 |
| 1-(1-enyl-palmitoyl)-GPC (P-16:0) | Lipid | Lysoplasmalogen | -0.967 | 0.0300316 | 0.0663336 |
| 1-(1-enyl-palmitoyl)-GPE (P-16:0) | Lipid | Lysoplasmalogen | -0.717 | 0.0320513 | 0.0694241 |
| 5-dodecenoate (12:1n7) | Lipid | Medium Chain Fatty Acid | 0.661 | 0.0085506 | 0.0306916 |
| mevalonate | Lipid | Mevalonate Metabolism | 3.873 | 0.0131865 | 0.0392005 |
| mevalonolactone | Lipid | Mevalonate Metabolism | 4.273 | 0.0067497 | 0.0264987 |
| 1-linoleoylglycerol (18:2) | Lipid | Monoacylglycerol | -0.738 | 0.0328247 | 0.0698943 |
| 2-linoleoylglycerol (18:2) | Lipid | Monoacylglycerol | -0.719 | 0.0293975 | 0.0655599 |
| 1-docosahexaenoylglycerol (22:6) | Lipid | Monoacylglycerol | 1.934 | 0.0323474 | 0.0696651 |
| 1-oleoyl-2-docosahexaenoyl-GPC (18:1/22:6) | Lipid | Phosphatidylcholine (PC) | 2.205 | 0.0437788 | 0.0808813 |
| 1,2-dioleoyl-GPE (18:1/18:1) | Lipid | Phosphatidylethanolamine (PE) | -3.001 | 0.0014861 | 0.0103724 |
| 1-palmitoyl-2-linoleoyl-GPE (16:0/18:2) | Lipid | Phosphatidylethanolamine (PE) | -2.997 | 0.000324 | 0.0049987 |
| 1-stearoyl-2-linoleoyl-GPE (18:0/18:2) | Lipid | Phosphatidylethanolamine (PE) | -2.414 | 0.0010382 | 0.0088382 |
| 1,2-dilinoleoyl-GPE (18:2/18:2) | Lipid | Phosphatidylethanolamine (PE) | -2.4 | 5E-07 | 6.49E-05 |
| 1-stearoyl-2-oleoyl-GPE (18:0/18:1) | Lipid | Phosphatidylethanolamine (PE) | -2.223 | 0.0008915 | 0.0086152 |
| 1-palmitoyl-2-oleoyl-GPE (16:0/18:1) | Lipid | Phosphatidylethanolamine (PE) | -2.22 | 0.0003039 | 0.0049987 |
| 1-palmitoyl-2-arachidonoyl-GPE (16:0/20:4) | Lipid | Phosphatidylethanolamine (PE) | -1.89 | 0.0035779 | 0.0175125 |
| 1-linoleoyl-2-arachidonoyl-GPE (18:2/20:4) | Lipid | Phosphatidylethanolamine (PE) | -1.762 | 0.0033873 | 0.0170967 |
| 1-palmitoyl-2-stearoyl-GPE (16:0/18:0) | Lipid | Phosphatidylethanolamine (PE) | -1.747 | 0.0004396 | 0.0056855 |
| 1-stearoyl-2-arachidonoyl-GPE (18:0/20:4) | Lipid | Phosphatidylethanolamine (PE) | -1.393 | 0.0047716 | 0.0213603 |
| 1-palmitoyl-2-oleoyl-GPI (16:0/18:1) | Lipid | Phosphatidylinositol (PI) | -0.932 | 0.0041327 | 0.0194697 |
| 1-palmitoyl-2-linoleoyl-GPI (16:0/18:2) | Lipid | Phosphatidylinositol (PI) | -0.921 | 0.0031326 | 0.0168666 |
| 1,2-dioleoyl-GPI (18:1/18:1) | Lipid | Phosphatidylinositol (PI) | -0.646 | 0.0131069 | 0.0392005 |
| phosphoethanolamine | Lipid | Phospholipid Metabolism | -1.609 | 0.0162399 | 0.0446763 |
| glycerophosphoinositol | Lipid | Phospholipid Metabolism | -1.578 | 0.0063793 | 0.0253085 |
| glycerophosphoethanolamine | Lipid | Phospholipid Metabolism | -1.518 | 0.0001479 | 0.0039818 |
| 1-(1-enyl-palmitoyl)-2-linoleoyl-GPC (P-16:0/18:2) | Lipid | Plasmalogen | -1.541 | 0.0033887 | 0.0170967 |
| 1-(1-enyl-palmitoyl)-2-palmitoleoyl-GPC (P-16:0/16:1) | Lipid | Plasmalogen | -1.141 | 0.0004425 | 0.0056855 |
| 1-(1-enyl-palmitoyl)-2-oleoyl-GPC (P-16:0/18:1) | Lipid | Plasmalogen | -1.008 | 0.0300964 | 0.0663336 |
| 1-(1-enyl-palmitoyl)-2-arachidonoyl-GPE (P-16:0/20:4) | Lipid | Plasmalogen | -0.836 | 0.0117746 | 0.0384476 |
| 1-(1-enyl-stearoyl)-2-oleoyl-GPE (P-18:0/18:1) | Lipid | Plasmalogen | -0.592 | 0.0257012 | 0.0616976 |
| linoleate (18:2n6) | Lipid | Polyunsaturated Fatty Acid (n3 and n6) | 0.919 | 0.0410142 | 0.0776776 |
| dihomo-linoleate (20:2n6) | Lipid | Polyunsaturated Fatty Acid (n3 and n6) | 1.466 | 0.0127525 | 0.0392005 |
| docosatrienoate (22:3n3) | Lipid | Polyunsaturated Fatty Acid (n3 and n6) | 1.507 | 0.0137274 | 0.0401065 |
| linolenate [alpha or gamma; (18:3n3 or 6)] | Lipid | Polyunsaturated Fatty Acid (n3 and n6) | 2.029 | 0.0499665 | 0.0867101 |
| hexadecatrienoate (16:3n3) | Lipid | Polyunsaturated Fatty Acid (n3 and n6) | 2.326 | 0.0292369 | 0.0655599 |
| docosapentaenoate (n3 DPA; 22:5n3) | Lipid | Polyunsaturated Fatty Acid (n3 and n6) | 2.575 | 0.0086514 | 0.0307607 |
| stearidonate (18:4n3) | Lipid | Polyunsaturated Fatty Acid (n3 and n6) | 3.255 | 0.0374243 | 0.0753417 |
| tauro-beta-muricholate | Lipid | Primary Bile Acid Metabolism | -3.217 | 9E-05 | 0.0034118 |
| tauroursodeoxycholate | Lipid | Secondary Bile Acid Metabolism | -2.055 | 0.012427 | 0.0391096 |
| taurolithocholate | Lipid | Secondary Bile Acid Metabolism | -1.668 | 0.004574 | 0.0207698 |
| taurolithocholate 3-sulfate | Lipid | Secondary Bile Acid Metabolism | -1.435 | 0.0397037 | 0.0771336 |
| ursodeoxycholate | Lipid | Secondary Bile Acid Metabolism | -1.411 | 0.0178718 | 0.0475679 |
| taurodeoxycholate | Lipid | Secondary Bile Acid Metabolism | -1.348 | 0.0494537 | 0.0866957 |
| sphingosine | Lipid | Sphingolipid Metabolism | -1.299 | 0.0057623 | 0.0236061 |
| sphinganine | Lipid | Sphingolipid Metabolism | -1.171 | 0.0003448 | 0.0049987 |
| sphinganine-1-phosphate | Lipid | Sphingolipid Metabolism | -1.086 | 0.0014827 | 0.0103724 |
| sphingadienine | Lipid | Sphingolipid Metabolism | -1.042 | 0.0032233 | 0.0168728 |
| N-palmitoyl-sphinganine (d18:0/16:0) | Lipid | Sphingolipid Metabolism | -0.87 | 0.0376671 | 0.0753417 |
| sphingomyelin (d18:2/21:0, d16:2/23:0) | Lipid | Sphingolipid Metabolism | -0.725 | 0.0226941 | 0.0570211 |
| phytosphingosine | Lipid | Sphingolipid Metabolism | -0.609 | 0.0270009 | 0.0632072 |
| behenoyl sphingomyelin (d18:1/22:0) | Lipid | Sphingolipid Metabolism | -0.605 | 0.0123929 | 0.0391096 |
| sphingomyelin (d18:2/14:0, d18:1/14:1) | Lipid | Sphingolipid Metabolism | 0.784 | 0.0120645 | 0.0388632 |
| sphingomyelin (d18:0/18:0, d19:0/17:0) | Lipid | Sphingolipid Metabolism | 0.797 | 0.0016094 | 0.01085 |
| myristoyl dihydrosphingomyelin (d18:0/14:0) | Lipid | Sphingolipid Metabolism | 1.008 | 0.000726 | 0.0075584 |
| sphingomyelin (d18:1/20:2, d18:2/20:1, d16:1/22:2) | Lipid | Sphingolipid Metabolism | 1.271 | 0.0129496 | 0.0392005 |

STable 1: log2FC, Log2 fold-change; FDR, false discovery rate. Some isomeric compounds were measured, but the different isomeric species cannot be be distinguished using untargeted UPLC-MS/MS. These different isomers are represented by a “[1]” or “[2]” after the biochical names.

Supplemental Table 2: Significantly Altered Lipid Metabolites

| **Biochemical Name** | **Super-Pathway** | **Sub-Pathway** | **log2FC** | **P-Value** | **FDR** |
| --- | --- | --- | --- | --- | --- |
| N-acetylglutamate | Amino Acid | Glutamate Metabolism | -1.528 | 0.00134 | 0.01011 |
| N-acetyl-aspartyl-glutamate (NAAG) | Amino Acid | Glutamate Metabolism | -0.749 | 0.00096 | 0.00881 |
| 2-hydroxybutyrate/2-hydroxyisobutyrate | Amino Acid | Glutathione Metabolism | -1.729 | 0.00116 | 0.00933 |
| N-acetylserine | Amino Acid | Glycine, Serine and Threonine Metabolism | -1.79 | 2E-05 | 0.00107 |
| glycine | Amino Acid | Glycine, Serine and Threonine Metabolism | -0.942 | 0.0031 | 0.01687 |
| 1-methylguanidine | Amino Acid | Guanidino and Acetamido Metabolism | -2.17 | 0.01046 | 0.03488 |
| 3-methylhistidine | Amino Acid | Histidine Metabolism | -0.858 | 0.0327 | 0.06989 |
| imidazole lactate | Amino Acid | Histidine Metabolism | 1.494 | 0.04848 | 0.08619 |
| isobutyrylglycine | Amino Acid | Leucine, Isoleucine and Valine Metabolism | -2.084 | 0.00436 | 0.02026 |
| alpha-hydroxyisovalerate | Amino Acid | Leucine, Isoleucine and Valine Metabolism | -1.196 | 0.00546 | 0.02295 |
| isovalerylglycine | Amino Acid | Leucine, Isoleucine and Valine Metabolism | -1.189 | 0.01252 | 0.03911 |
| N-acetylisoleucine | Amino Acid | Leucine, Isoleucine and Valine Metabolism | -0.842 | 0.01801 | 0.04757 |
| tiglylcarnitine (C5:1-DC) | Amino Acid | Leucine, Isoleucine and Valine Metabolism | 0.868 | 0.02847 | 0.06524 |
| 5-hydroxylysine | Amino Acid | Lysine Metabolism | -2.952 | 0.00622 | 0.02493 |
| pipecolate | Amino Acid | Lysine Metabolism | -1.804 | 8E-05 | 0.00341 |
| N6-acetyllysine | Amino Acid | Lysine Metabolism | -1.458 | 0.00107 | 0.00884 |
| N2-acetyllysine | Amino Acid | Lysine Metabolism | -1.17 | 0.00269 | 0.01537 |
| 6-oxopiperidine-2-carboxylate | Amino Acid | Lysine Metabolism | -0.845 | 0.00144 | 0.01037 |
| 2-aminoadipate | Amino Acid | Lysine Metabolism | -0.834 | 0.00344 | 0.0171 |
| lysine | Amino Acid | Lysine Metabolism | -0.809 | 0.02662 | 0.0627 |
| N6,N6,N6-trimethyllysine | Amino Acid | Lysine Metabolism | -0.807 | 0.00135 | 0.01011 |
| 5-aminovalerate | Amino Acid | Lysine Metabolism | 0.818 | 0.0181 | 0.04757 |
| N-acetylmethionine sulfoxide | Amino Acid | Methionine, Cysteine, SAM and Taurine Metabolism | -2.246 | 0.00441 | 0.02026 |
| hypotaurine | Amino Acid | Methionine, Cysteine, SAM and Taurine Metabolism | -1.406 | 0.00032 | 0.005 |
| S-adenosylhomocysteine (SAH) | Amino Acid | Methionine, Cysteine, SAM and Taurine Metabolism | -1.162 | 0.04269 | 0.07968 |
| methionine sulfoxide | Amino Acid | Methionine, Cysteine, SAM and Taurine Metabolism | -0.952 | 0.04054 | 0.07768 |
| methionine sulfone | Amino Acid | Methionine, Cysteine, SAM and Taurine Metabolism | -0.741 | 0.01582 | 0.0441 |
| cystine | Amino Acid | Methionine, Cysteine, SAM and Taurine Metabolism | 0.595 | 0.04034 | 0.07768 |
| alpha-ketobutyrate | Amino Acid | Methionine, Cysteine, SAM and Taurine Metabolism | 0.721 | 0.03581 | 0.07443 |
| cysteine s-sulfate | Amino Acid | Methionine, Cysteine, SAM and Taurine Metabolism | 2.654 | 0.01988 | 0.05027 |
| phenylpyruvate | Amino Acid | Phenylalanine Metabolism | -1.074 | 0.00023 | 0.005 |
| N-acetylputrescine | Amino Acid | Polyamine Metabolism | -0.983 | 0.02422 | 0.05856 |
| spermidine | Amino Acid | Polyamine Metabolism | -0.909 | 0.00765 | 0.029 |
| indolepropionate | Amino Acid | Tryptophan Metabolism | -1.428 | 0.0336 | 0.07114 |
| serotonin | Amino Acid | Tryptophan Metabolism | -1.078 | 0.0376 | 0.07534 |
| 3-methoxytyrosine | Amino Acid | Tyrosine Metabolism | -0.938 | 0.01899 | 0.04898 |
| 2-hydroxyphenylacetate | Amino Acid | Tyrosine Metabolism | -0.844 | 0.04083 | 0.07768 |
| 3-(4-hydroxyphenyl)lactate | Amino Acid | Tyrosine Metabolism | -0.782 | 0.02404 | 0.05856 |
| 3-methoxytyramine sulfate | Amino Acid | Tyrosine Metabolism | -0.711 | 0.01453 | 0.04147 |
| N-delta-acetylornithine | Amino Acid | Urea cycle; Arginine and Proline Metabolism | -1.829 | 0.00053 | 0.00618 |
| ornithine | Amino Acid | Urea cycle; Arginine and Proline Metabolism | -1.527 | 0.01405 | 0.04073 |
| N-methylproline | Amino Acid | Urea cycle; Arginine and Proline Metabolism | -1.342 | 0.04232 | 0.07968 |
| 2-oxoarginine* | Amino Acid | Urea cycle; Arginine and Proline Metabolism | -1.325 | 0.00797 | 0.02944 |
| N-acetylarginine | Amino Acid | Urea cycle; Arginine and Proline Metabolism | -1.316 | 0.00487 | 0.02136 |
| argininosuccinate | Amino Acid | Urea cycle; Arginine and Proline Metabolism | -1.102 | 0.02752 | 0.06364 |
| trans-4-hydroxyproline | Amino Acid | Urea cycle; Arginine and Proline Metabolism | -1.088 | 0.04777 | 0.08583 |
| N-monomethylarginine | Amino Acid | Urea cycle; Arginine and Proline Metabolism | -0.884 | 0.00183 | 0.01192 |
| proline | Amino Acid | Urea cycle; Arginine and Proline Metabolism | -0.782 | 0.02374 | 0.05856 |
| arginine | Amino Acid | Urea cycle; Arginine and Proline Metabolism | -0.647 | 0.02381 | 0.05856 |

STable 2: log2FC, Log2 fold-change; FDR, false discovery rate. Some isomeric compounds were measured, but the different isomeric species cannot be be distinguished using untargeted UPLC-MS/MS. These different isomers are represented by a “[1]” or “[2]” after the biochical names.
