# Supplementary figures and images for: Untargeted analysis of the serum metabolome in cats with exocrine pancreatic insufficiency
Source: PLoS One. 2021 Sep 30;16(9):e0257856. doi: 10.1371/journal.pone.0257856 (PMC8483406; doi:10.1371/journal.pone.0257856)

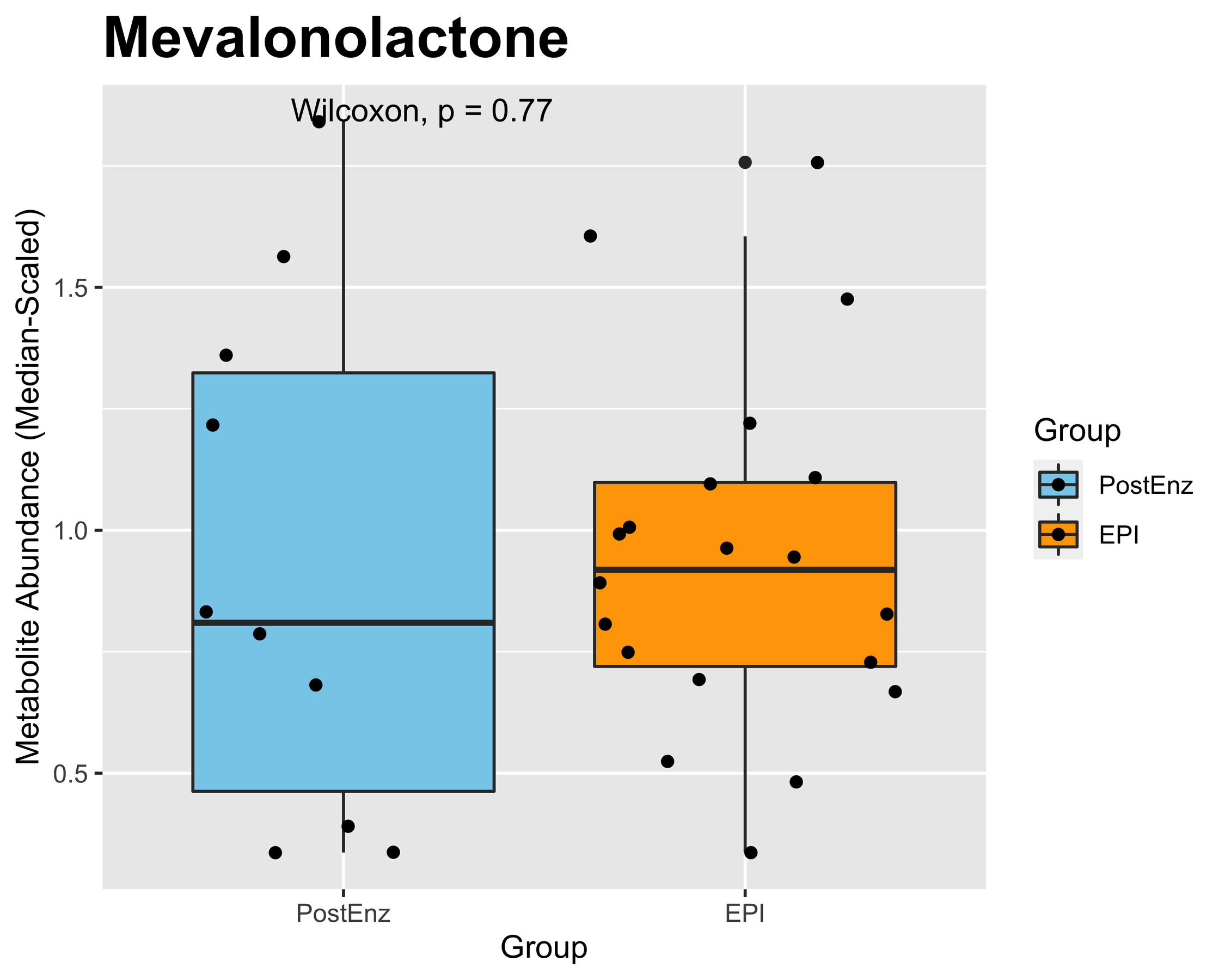

Supplement: S1 Fig — The wilxocon rank-sum test was used to compare the abundance of mevalonolactone in the sera of dogs with EPI and healthy dogs (PostEnz). Unlike cats with EPI, there is no significant difference in the abundance of mevalonolactone in the sera of dogs with EPI compared with healthy controls. (TIF) [file pone.0257856.s006.tif]
